# Integrative metagenomic and biochemical studies on rifamycin ADP-ribosyltransferases discovered in the sediment microbiome

**DOI:** 10.1038/s41598-018-30547-x

**Published:** 2018-08-14

**Authors:** Jae Hong Shin, Hyunuk Eom, Woon Ju Song, Mina Rho

**Affiliations:** 1 Department of Computer Science and Engineering, Hanyang University, Seoul, Korea; 2 Department of Chemistry, Seoul National University, Seoul, 08826 Korea; 3 Department of Biomedical Informatics, Hanyang University, Seoul, Korea

## Abstract

Antibiotic resistance is a serious and growing threat to human health. The environmental microbiome is a rich reservoir of resistomes, offering opportunities to discover new antibiotic resistance genes. Here we demonstrate an integrative approach of utilizing gene sequence and protein structural information to characterize unidentified genes that are responsible for the resistance to the action of rifamycin antibiotic rifampin, a first-line antimicrobial agent to treat tuberculosis. Biochemical characterization of four environmental metagenomic proteins indicates that they are adenosine diphosphate (ADP)-ribosyltransferases and effective in the development of resistance to FDA-approved rifamycins. Our analysis suggests that even a single residue with low sequence conservation plays an important role in regulating the degrees of antibiotic resistance. In addition to advancing our understanding of antibiotic resistomes, this work demonstrates the importance of an integrative approach to discover new metagenomic genes and decipher their biochemical functions.

## Introduction

Antibiotics are indispensable therapeutic agents for human health against infection from pathogenic bacteria. The emergence of resistance against antibiotics, however, has outpaced the discovery of new antibiotics. The Centers for Disease Control and Prevention (CDC) and the World Health Organization (WHO) declared that we are in the post-antibiotic era and are facing an antibiotic resistance crisis. This critical situation urges for immediate actions in politics, research, and health care systems^1^.

Tuberculosis is a widespread infectious disease, which is caused mostly by *Mycobacterium tuberculosis*^2,3^. Currently, nearly one-third of the global population is infected by the pathogenic bacteria. Rifampin, also known as rifampicin, belongs to the rifamycin family, and is a first-line antimicrobial agent against tuberculosis. Rifampin binds to the β subunit of DNA-dependent RNA polymerase, and inhibits the RNA synthesis of bacteria through steric occlusion^4,5^.

Since rifampin is an effective anti-tuberculosis agent, molecular mechanisms underlying the resistance to rifampin have been studied extensively^6–9^. A few mutations in bacterial RNA polymerase attenuate the binding affinity of rifampin^10,11^. Alternatively, rifampin can be modified by glycosylation^12^, adenosine diphosphate (ADP)-ribosylation^13^, monooxygenation^14^, or phosphorylation^7^, which also results in decreased binding affinity to RNA polymerase. The glycosyltransferase Rgt1438 confers rifampin resistance by converting rifampin to 23-O-glu-rifampin^12^. Rifampin ADP-ribosyltransferases (Arr) attach an ADP-ribosyl group to the hydroxyl group at the C21 position of rifampin to form ADP-ribosylrifampin (Additional file 1: Scheme 1a)^13,15–17^. Rifampin phosphotransferase (RPH), recently identified from *Listeria monocytogenes*^7^, is a kinase that modifies rifampin to phospho-rifampin at the C21 position^7,18^. The diversity in the chemical origins implies that antibiotic resistance can readily be acquired by multiple organisms. Therefore, antibiotic resistance genes and related proteins should be explored as immediate targets of research to understand comprehensive antibiotic resistomes^19,20^.

With the advances in sequencing technology, metagenomic approaches have been applied to identify antibiotic resistance genes from environmental samples^21–24^. Since many antibiotics originate from secondary metabolites of bacteria^25,26^, resistance determinants are ubiquitously found in microorganisms, even in ancient environments^27–31^. As such, reservoirs of resistance determinants have been investigated to profile abundant resistance genes and their effects on microbiome composition^32^. Nesme *et al*. analyzed antibiotic resistance gene determinants from 71 environmental shotgun metagenomic samples from a global environment survey^33^. Li *et al*. applied network analysis to identify 18 types and 260 subtypes of antibiotic resistance genes^34^.

In order to deduce the functions of particular genes and proteins, sequence similarity has been widely applied as an important measure. For example, resistome profiling^35^ or functional metagenomics^36,37^ have been applied to reveal the genes related to the antibiotic resistances. Studies of conserved residues alone, however, might not be sufficient for predicting the function of the genes^38^. An inherent limitation of sequence similarity-based approaches becomes more pronounced in determining the functions of newly discovered genes^39^. Recently, Balskus *et al*. have developed a profiling method by combining protein sequence similarity network analysis with quantitative metagenomics to discover an unidentified enzyme, suggesting that additional information can greatly improve the quality of metagenomic data^40^.

Within this context, we have developed an integrative approach of combining sequence analysis and molecular interaction analysis of the protein structure with highest sequence similarity. Potential *arr* genes (*arr-wd)*, obtained from the sediment microbiome, were analyzed to locate conserved functional sites. As an experimental validation of our computational prediction, *in vitro* and *in vivo* resistance activity assays were carried out with rifamycins to assess the ADP-ribosyltransferase activity of expressed proteins. This work represents an example of using microbiome sequence data to discover proteins related to the antibiotic resistance, to construct detailed three-dimensional models to extract functionally important structural features, and to validate computational predictions by biochemical experimental studies.

## Results and Discussion

### Novel rifamycin ADP-ribosyltransferase genes in the sediment microbiome

**Figure 1:**
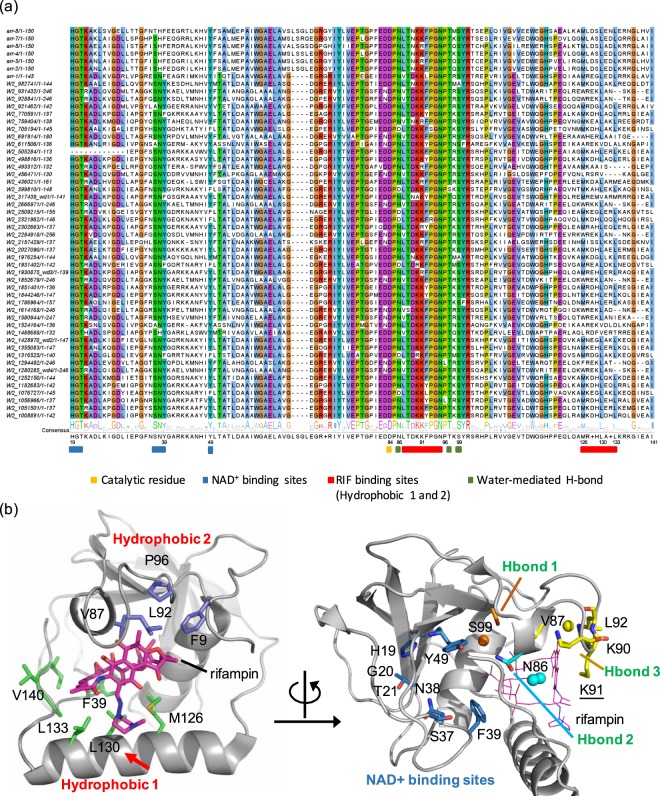
Integrated analysis of rifamycin ADP-ribosyltransferase (Arr). (**a**) The sequence conservation of rifamycin ADP-ribosyltransferase (Arr). The residues that are conserved more than 78% of the sequences are highlighted based on the clustalw color scheme. The genes selected for experimental validation are shown as wd1-wd4. (**b**) Proposed key residues and their molecular interactions in the rifampin-binding sites of Arr-*ms* structure (PDB 2HW2). In Arr-*ms*, the variable X residue in M[R|K][D|E]XL motif is Gly129, and its position is highlighted with a red arrow. Water molecules are depicted with spheres.

A total of 45 rifamycin ADP-ribosyl transferase (*arr*) homologous genes (*arr-wd*) were obtained from the sediment microbiome with the query of seven *arr* genes annotated in the comprehensive antibiotic resistance database (CARD) (Additional file 1: Table S1)^41,42^. The similarity between the seven known *arr* genes and 45 *arr-wd* genes ranges from 48.23 to 71.01%. These 45 putative *arr* genes were aligned with the seven known *arr* genes to find the conserved functional regions in the amino acid sequences (Fig. 1a). ADP-ribosyltransferases exhibit multiple conserved motifs that are presumably related to rifampin binding and nicotinamide adenine dinucleotide (NAD^+^) binding through hydrogen bonding, hydrophobic interactions, and cation-pi interactions (Fig. 1b). Our results include the rifampin and NAD^+^ binding sites reported in the previous studies of *arr-ms* from *Mycobacterium smegmatis*, *arr-sc* from *S. coelicolor*, and *arr-2* and homologous genes (*arr*-3, *arr-*4, *arr-*5, *arr*-7, and *arr*-8) from several gram-negative pathogenic bacteria^13,43–46^. In particular, H19 and Y49, which were identified for NAD^+^ binding^13^, were highly conserved in our alignment.

### Structure-guided conservation analysis

It has been proposed that NAD^+^ binding sites in ADP-ribosyltransferases are formed by a long flexible loop between β1 and β3 strands (Fig. 1b; Additional file 1: Figure S1), and several residues constituting the loop are essential for the function of Arr^13^. A HGT motif (H19, G20, and T21 in Arr-*ms*) at the end of β1 strand and a tyrosine (Y49 in Arr-*ms*) in β3 strand are spatially adjacent to an aspartate (D84). Site-directed mutagenesis studies have demonstrated that these amino acids, H19, Y49, and D84 in Arr-*ms*, are critical for the catalysis through hydrogen-bonding interactions with NAD^+13^. Indeed, these residues are highly conserved in the putative rifampin ADP-ribosyltransferase genes of the current study: 98.0% for H19, 87.0% for Y49, and 100% for D84 (Fig. 1a).

**Figure 2:**
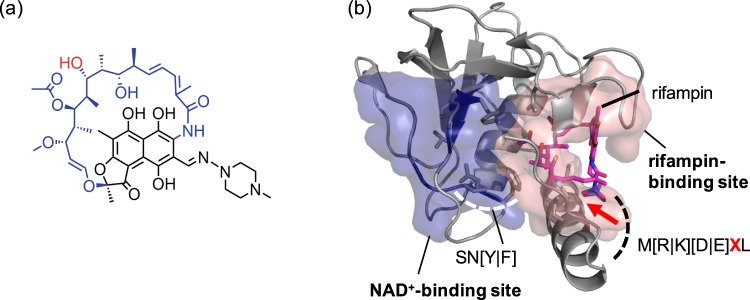
Structural analysis of rifamycin ADP-ribosyltransferase (Arr). (**a**) A structure of rifampin. The aliphatic ansa chain and the site of modification by ADP-ribosyltransferase is colored in blue and red, respectively. (**b**) Two substrate-binding pockets depicted in the structure of rifamycin ADP-ribosyltransferase from arr-*ms* (PDB 2HW2). The variable X residue in M[R|K][D|E]XL motif is  highlighted with a red arrow.

In addition to the HGT motif, we found that SN[Y|F] motif in the flexible loop region between β2 and β3 strands (Figs 1a and 2b; Additional file 1: Figure S1) is highly conserved (92.0% for S, 87.0% for N, 79% for Y, 21% for F). Although the X-ray crystal structure of Arr complexed with NAD^+^ is not available, a docking simulation indicates that the SN[Y|F] motif is likely to be located at the entrance to the active site, providing the shape and/or charge complementarity to the sugar-phosphate group of NAD^+^ (Additional file 1: Figure S2). Presumably, such non-covalent interaction with HGT and SN[Y|F] motifs polarizes the nicotinamide portion of NAD^+^ to help generate the oxocarbenium intermediate.

The X-ray crystal structure of Arr-*ms* (PDB ID 2HW2)^13^ displays a large binding pocket to accommodate rifampin (solvent accessible surface area = 621.95 Å^2^), comprised of amino acid residues from α1 and α2 helices and a loop between β5 and β6 strands. We categorized the residues into four types, depending on their molecular interactions, *hydrophobic 1*, *hydrophobic 2*, *cation-pi*, and *water-mediated hydrogen bond* (Fig. 1b). High conservation was indeed observed in the *arr* genes that we predicted from the metagenomic data. All key amino acid residues comprising the rifampin-binding site were well-conserved (Fig. 1a).

The *hydrophobic 1* region is a main hydrophobic pocket for rifampin-binding. It is comprised of hydrophobic bulky residues, F39, M126, L130, L133, A139, and I141 in Arr-*ms* (Fig. 1b). The three residues M126, L130, and L133, surrounding the piperazine ring portion of rifampin, are located in helix α2. The piperazine ring of rifampin is likely to be inserted between L133 and M126 by a hydrophobic interaction. L130 also possesses hydrophobic characteristics and is highly conserved in our metagenome sequences [L(88.0%)|V(10.0%)|I(2.0%)], thus contributing to the formation of hydrophobic pocket that is positioned close to the piperazine ring. In helix α2, M[R|K][D|E]XL residues seem to be essential for rifampin-binding; they are highly conserved for both the reference and metagenome sample sequences. Since the methionine, the fourth amino acid (labeled as X), and two leucine residues are positioned toward the pocket in Arr-*ms*, a variation of these key residues would regulate the binding affinity and/or activities. In the aliphatic ansa chain of rifampin (Fig. 2a), two methyl groups (C31 and C32) and diene units are surrounded by F39, A139, and I141, which allow hydrophobic interactions.

The *hydrophobic 2* region is a shallow and rather surface-exposed pocket. This region consists of F9, V87, L92, and P96 (Fig. 1b). While the residues F9, L92, and P96 are positioned in the loops, and directly exposed to the aqueous environment, they are spatially close to engage in hydrophobic interaction with a methyl group (C14) of rifampin. The residues L92 and P96 are located in the loop structure between β5 and β6 strands, and are part of the *rifampin binding loop*^13^. The residue V87 is positioned between β5 and β6 strands, generating a small α-helix structure.

K91 is located in the flexible loop structure between β5 and β6 strands (Fig. 1b). The residue is positioned above the naphthoquinone ring portion of rifampin, and the positively charged ε-ammonium (NH_3_^+^) of K91 makes a cation–pi contact. The residue is highly conserved in most of the Arr protein, constituting 85% of lysine and 15% of arginine. This spatial arrangement effectively shields the aromatic rings from exposure to the aqueous environment. The adjacent amino acids are also highly conserved as [L|V]TK[K|R]P: 73.0% for L; 98.0% for T; 94.0% for K; 85% for K; 96.0% for P.

In addition to the rifampin–Arr-*ms* interactions discussed above, we also observe three *water-mediated hydrogen bonds*, which might be necessary for rifampin binding (Fig. 1b, Additional file 1: Figure S1). In the rifampin binding loop, a water molecule (numbered as 1226) forms hydrogen bonding networks between a hydroxyl group (O2, 3.07 Å) of the rifampin naphthoquinone ring and backbone amide groups from V87, T88, K90, and L92 residues (2.89–3.04 Å). As discussed above, these residues are also involved in the hydrophobic interaction with rifampin, which might further contribute to the binding affinity. Another water molecule (numbered as 1335) mediates hydrogen bonding between the carbonyl oxygen atom of the O-acetyl (O8, 3.26 Å) group of rifampin and the hydroxyl group of S99 (OG, 2.77 Å). In addition, two water molecules (numbered as 1230 and 1381) form hydrogen bonding networks between N86, V140, and the N–H group of rifampin. These conserved residues are represented as DPN[V|L]. Here, D84 denotes the catalytic residue for the ADP-ribosylation function in Arr-*ms*^13^.

### Phylogenetic relationship of arr genes

**Figure 3:**
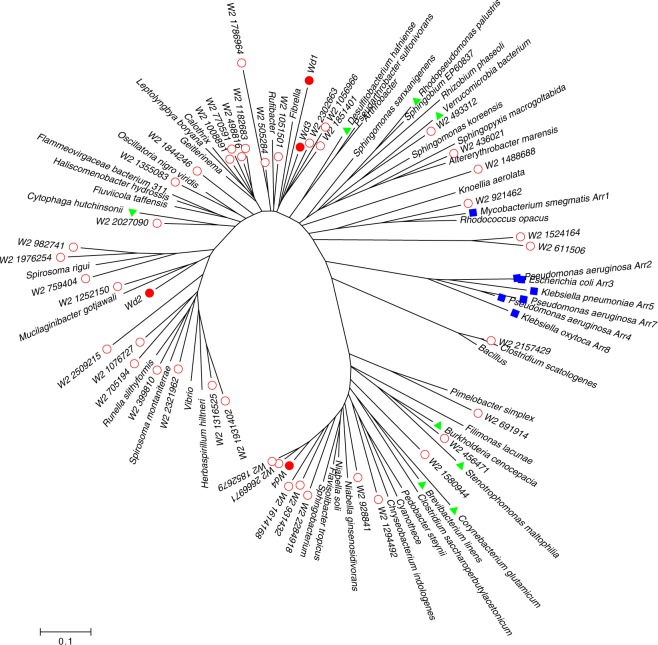
A phylogenetic tree of the metagenomic arr genes and well-known *arr* genes. Red circles denote arr genes collected from the metagenomic sample in this work. Red filled circles denote arr genes validated experimentally in this work. Blue rectangles denote previously known and experimentally validated arr genes. Green triangles denote *arr* genes referenced previously but not validated experimentally.

In order to find the phylogenetic relationship of the newly found *arr* genes, *arr-wd*, with the known genes, a neighbor-joining tree was built. In the phylogenetic tree, four arr-wd genes (*arr-wd*1-4) experimentally validated in this study were analyzed with the known *arr* genes (Fig. 3). In addition to *arr-ms* and known *arr* genes from gram-negative bacteria (*arr-2*, *arr-3, arr-4*, *arr-5*, *arr-7*, and *arr-8*; denoted as blue filled rectangles in Fig. 3), *arr-wd1, arr-wd2*, *arr-wd3, and arr-wd4* (denoted as red filled circles in Fig. 3) are placed with the homologous *arr* genes identified from the complete genomes. In particular, *arr-wd1, arr-wd2*, and *arr-wd3* are clustered with the *arr* genes from *Rhodopseudomonas palustris*, *Desulfitobacterium hafniense*, *Verrucomicrobium spinosum*, and *Cytophaga hutchinsonii*, which were used in the phylogenetic tree of a previous study^13^. On the other hand, *arr-wd4* is clustered with the genes from *Brevibacterium linens, Corynebacterium glutamicum, Burkholderia cenocepacia*, and *Stenotrophomonas maltophilia*. While the previous study showed a wide distribution of *arr* genes in the phylogenetic tree^13^, our study expanded the phylogenetic tree by incorporating diverse *arr* genes from an environmental sample and validating them experimentally.

To find the origins of the microbial genomes, the *arr-wd* genes were investigated by using homology search. We found that the *arr* gene *W2_2157429* shows the highest homology (98.54% protein similarity) to *Clostridium ljungdahlii*. A total of 13 out of 45 *arr-wd* genes show high homology (>80% protein similarity) to the known species (Additional file 1: Table S2), implying that *arr-wd* like genes might be prevalent in various organisms, although they are yet to be annotated.

### Biochemical analysis of representative arr genes

**Figure 4:**
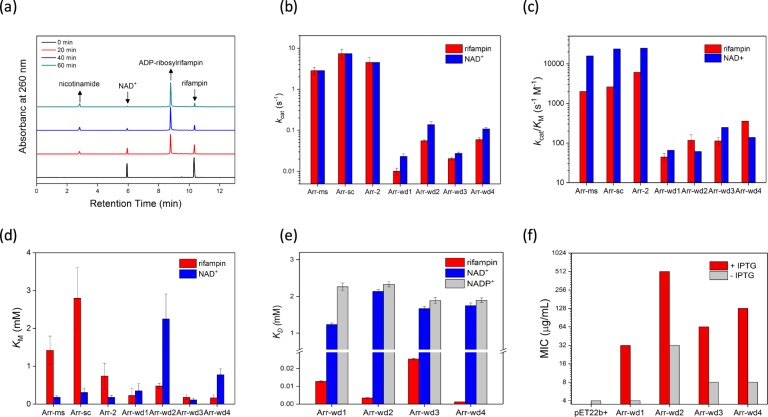
Biochemical analysis of Arr-wd 1–4 proteins. (**a**) A representative HPLC trace of rifampin ADP-ribosylation by Arr-wd proteins. (**b–d**) Steady-state ADP-ribosyltransferase activities of Arr-wd proteins together with the reported values of Arr proteins. (**b**) Turnover numbers, *k*_cat_ (s^−1^) (**c**) Catalytic efficiencies, *k*_cat_/*K*_M_ (s^−1^ M^−1^) (**d**) Michaelis constants, *K*_M_ (mM) (**e**) Dissociation constants, *K*_D_ (mM) (**f**) MIC values (μg/mL). Values reported in (**b**–**d**) and (**e**) are derived from the fit to the Michaelis-Menten equation and Stern-Volmer equation, respectively.

To evaluate whether the newly discovered genes display ADP-ribosyltransferase activity, we selected four metagenomic genes (arr-wd 1-4) and expressed the corresponding proteins (Additional file 1: Table S3 and Figure S3). All four putative *arr* genes possess the conservation of the essential functional residues (Fig. 1a), comprised of NAD^+^ and rifampin-binding pockets (Fig. 2), and exhibit rifampin ADP-ribosyltransferase activities (Fig. 4; Additional file 1: Tables S5–S6 and Figure S4). The activities are relatively lower than the previously characterized *arr* genes^13^ by about 1–2 orders of magnitude in both turnover number (*k*_cat_) and catalytic efficiency (*k*_cat_/*K*_M_). The data, however, definitively indicate that all Arr-wd proteins discovered herein are ADP-ribosyltransferases that modify rifampin upon the reaction with NAD^+^, and the metagenomic sequences can be denoted as *arr* genes that induce the resistance of rifampin. Interestingly, Michaelis constant (*K*_M_) and the dissociation constant (*K*_D_) of the Arr-wd proteins for rifampin are significantly lower than those of the previously reported Arr proteins (Fig. 4d,e; Additional file 1: Table S7). These results indicate that Arr-wd proteins exhibit tighter binding to the rifampin than other Arr proteins, and the arr-wd genes could start to develop resistance even at low concentration levels of antibiotics when sufficient *k*_cat_ is provided.

The binding constants for NAD^+^ and rifampin were also measured with Arr-wd3 protein without the His-tag (Arr-wd3 + Δ6His) (Additional file 1: Table S7). The protein yields only slightly perturbed values, indicating that Arr-wd proteins expressed with the His-tag are valid for evaluating the biochemical activities.

The *in vivo* activities of the proteins were determined by the minimum inhibitory concentration (MIC) values (Fig. 4f; Additional file 1: Table S9). Their MIC values are slightly lower than other arr proteins, presumably due to their lower *in vitro* activities of Arr-wd proteins. Their MIC values, however, are considerably higher than the background level, again suggesting that the *arr-wd* genes can inactivate the antibiotics in considerable rates. The ADP-ribosyltransferase activities measured both *in vitro* and *in vivo* conditions, therefore, convincingly demonstrate that we have discovered a novel set of *arr* genes from environmental metagenomes, and have predicted their biochemical function.

### Biochemical analysis of NAD^+^ binding sites

Our structure-guided conservation analysis and *in vitro* activity assay described above suggest that putative Arr-wd proteins are likely to have structurally well-defined NAD^+^ and rifampin-binding sites, which are essential for the dedicated chemical reaction. When NAD^+^ was replaced with nicotinamide adenine dinucleotide phosphate (NADP^+^), NADH, and NADPH, no ADP-ribosyltransferase activity was observed, indicating that Arr-wd proteins exhibit selective interaction with NAD^+^ over other analogous cofactors.

As described above, the SN[Y|F] motif is highly conserved, composing the putative NAD^+^-binding site: SNF in Arr-wd1 and SNY in Arr-wd2-4. The variation of the third residue between Phe and Tyr seems to be insignificant because there is no clear correlation between the sequence and binding affinity (*K*_D_) or ADP-ribosyltransferase activity (Additional file 1: Tables S6–S7 and Figures S4–S5). Therefore, only the hydrophobic interactions with the benzene ring of the side chain seems to be sufficient for retaining the interaction with NAD^+^. Since the Tyr or Phe residue is located at the intersection of NAD^+^ and rifampin-binding sites, it is possible that this hydrophobic residue also protects the oxocarbenium ion from other side reactions (Additional file 1: Scheme S1b and Figure S2).

Our sequence analysis also demonstrate that a large portion of the previously reported genes have SH[F], instead of the SN[Y|F] motif, with histidine (H) rather than asparagine (N) in the second position. To evaluate whether the sequence variation is related to the action of rifampin ADP-ribosyltransferase, we carried out site-directed mutagenesis of one of the metagenomes, arr-wd3. The N34H mutant of Arr-wd3 yields 6-fold lower *k*_cat_/*K*_M_ and slightly elevated *K*_D_ values than Arr-wd3 (Additional file 1: Tables S7–S8). The results imply that the residue is involved in NAD^+^-binding and/or positioning, and the N34H mutation perturbs the interactions with NAD^+^. Interestingly, the *K*_D_ value for rifampin has been also altered by the N34H mutation, suggesting that the mutation might have allosterically influenced the thermodynamic parameters associated with the rifampin-binding event.

### Biochemical analysis of rifampin-binding sites

Steady-state activity data demonstrate that the Arr-wd proteins are kinetically competent in rifampin ADP-ribosylation, although their activities are lower than those of the previously reported Arr proteins. Notably, however, the Arr-wd proteins exhibit considerably lower *K*_M_ and *K*_D_ values for rifampin, implicating that Arr-wd proteins can interact with rifampin in a thermodynamically favorable manner.

**Figure 5:**
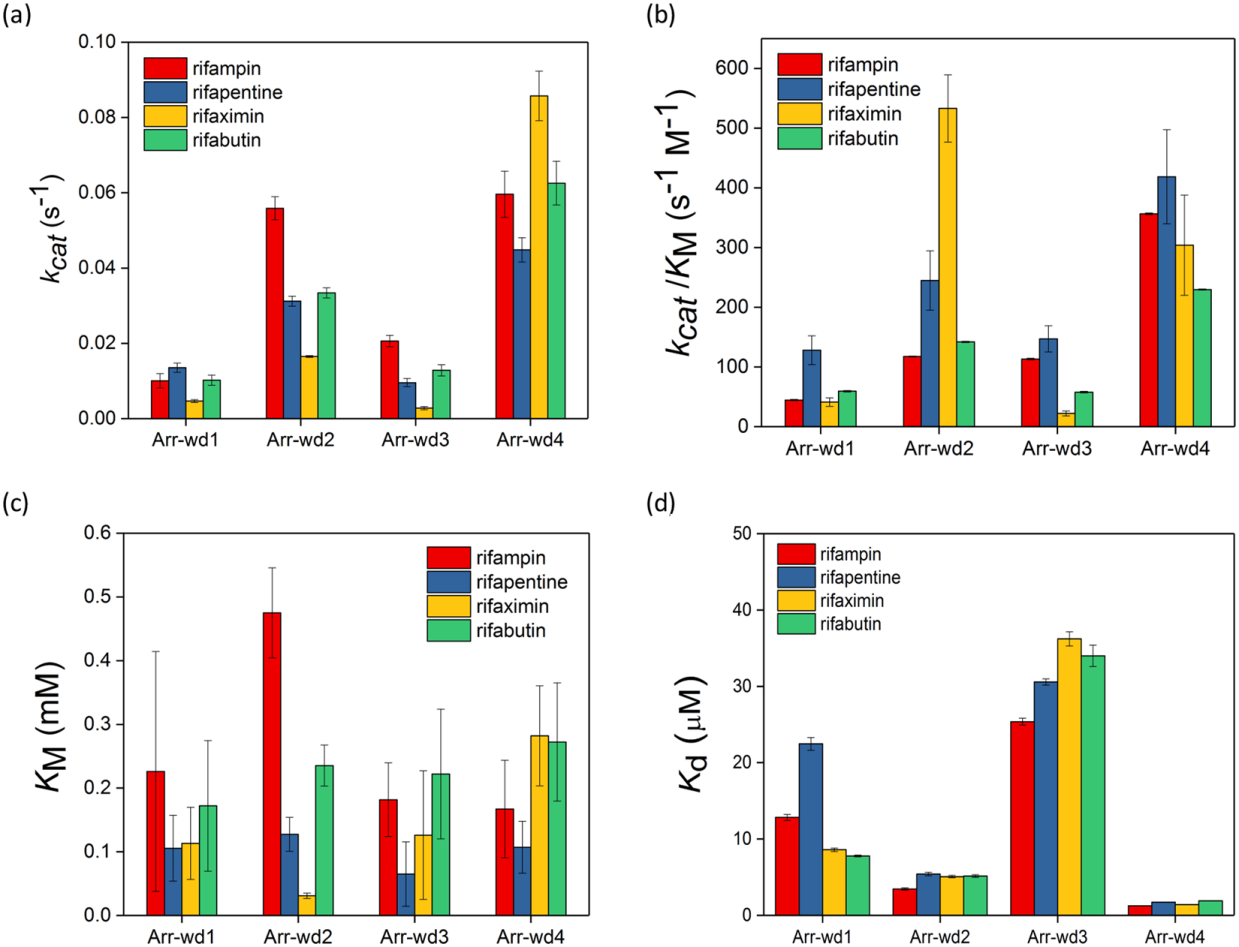
Kinetic and thermodynamic parameters of Arr-wd proteins from the steady-state reactions with rifampin derivatives, rifampin, rifapentine, rifaximin, and rifabutin. (**a**) Turnover numbers, *k*_cat_ (s^−1^) (**b**) Catalytic efficiencies, *k*_cat_/*K*_M_ (s^−1^ M^−1^) (**c**) Michaelis constants, *K*_M_ (mM) (**d**) Dissociation constants, *K*_D_ (μM). Values reported in (**a**–**c**) and (**d**) are derived from the fit to the Michaelis-Menten equation and Stern-Volmer equation, respectively.

We evaluated whether the proteins can react with other semisynthetic rifampin analogues that are clinically approved and prescribed as drugs, such as rifapentine, rifaximin, and rifabutin (Additional file 1: Table S6 and Figures S6 and S7)^47^. All Arr-wd proteins react with these analogues to afford the corresponding ADP-ribosylated products (Table S5). However, their kinetic parameters are markedly altered depending on the substrates (Fig. 5), implying that modified chemical structure at the C8 position of the naphthoquinone core significantly changes the degrees of interaction with Arr-wd proteins. In general, Arr-wd2 and Arr-wd4 exhibit higher steady-state activity than Arr-wd1 and Arr-wd3. But Arr-wd2 and Arr-wd3 exhibit noticeably high and low *k*_cat_/*K*_M_ for rifaximin, respectively, implying that specific interaction with the protein environment is operative in the ADP-ribosylation of the rifamycins. In contrast, rifapentine seems to be easily modified by all Arr-wd 1–4 proteins, indicating that the substrate is more promiscuous than other rifampin analogues.

To evaluate whether the altered catalytic activities are originated from the perturbation in the initial step of the reaction, i.e. association of the substrate to the protein, we measured the rifampin binding constants by monitoring intrinsic tryptophan fluorescence. The *K*_D_ values are in the order of Arr-wd4 ≈ Arr-wd2 < Arr-wd1 < Arr-wd3. This trend is similar to the inverse of the *k*_cat_/*K*_M_, implying that substrate binding is likely to be the part of the rate-determining steps in catalysis. However, no significant difference was observed between the *K*_D_ values of the rifampin analogues. Presumably, fluorescence changes of the tryptophan residue might not be sufficiently sensitive to differentiate the structural changes of the proteins that entail ADP-ribosyl transfer during steady-state reactions.

The chemical origin of the different activities across rifampin analogues is yet to be delineated. We tentatively conclude that amino acid residues comprising the rifampin-binding sites could be responsible for this differentiation. As described above, the α2-helix composed of M[R|K][D|E]XL residues at the C-terminus makes direct contact with rifampin (Figs 1b and 2b), and is conserved throughout the metagenomic sequences (Fig. 1a). Although the fourth residue X is one of the least conserved residues in our metagenome sequences as G(15.56%), K(17.78%), H(40%), A(8.89%), R(4.44%), K(6.67%), M(2.22%), S(2.22%), and F(2.22%), our structural analysis suggests that it is located in proximity to the complexed rifampin, directly pointing towards the substituent at the C8 position. Therefore, we suppose that it may play an important role in determining the ADP-ribosyltransferase activity.

### Site-directed mutagenesis of rifampin-binding sites

**Figure 6:**
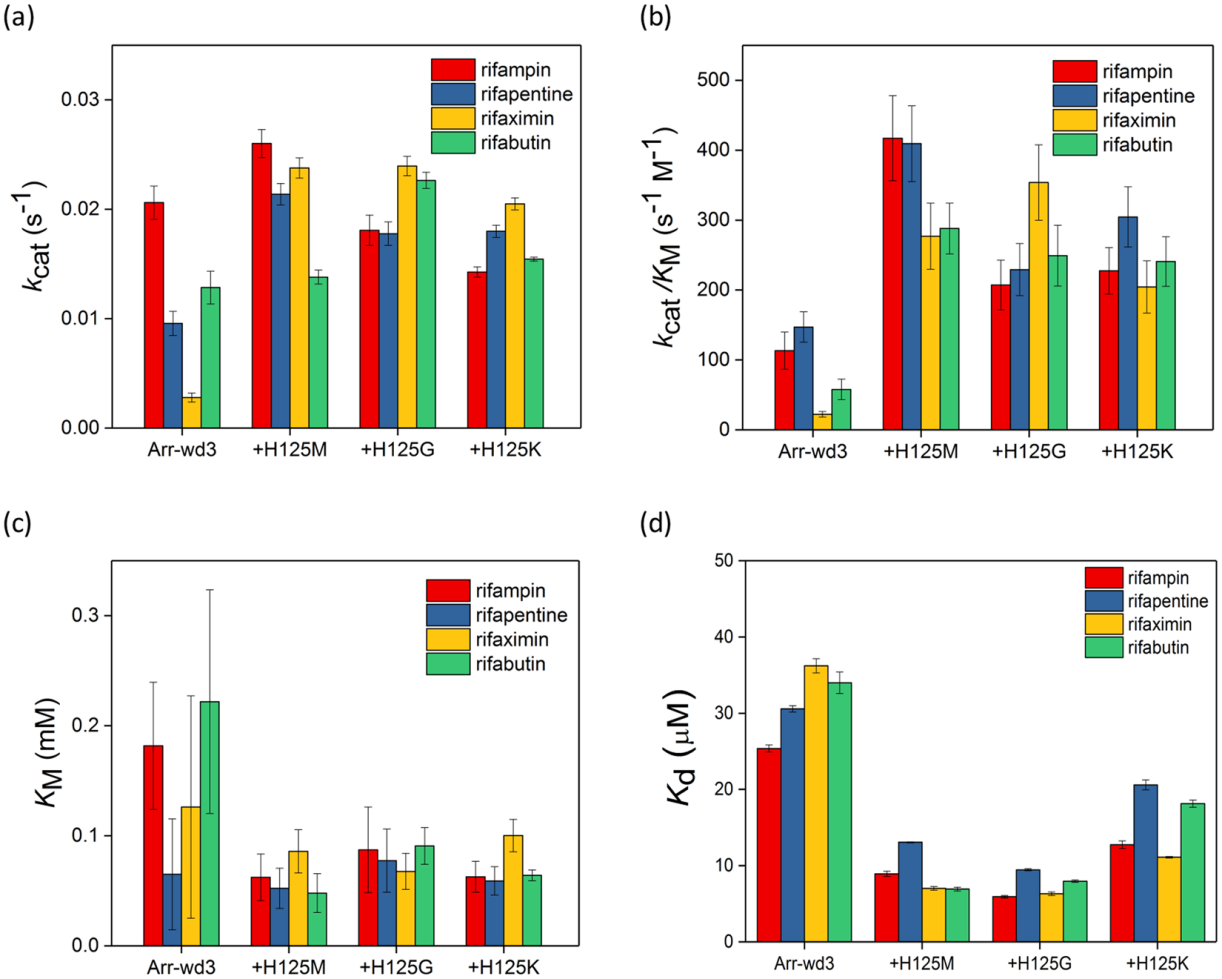
Kinetic and thermodynamic parameters of Arr-wd3 H125 variants from the steady-state reactions with rifampin derivatives. (**a**) Turnover numbers, *k*_cat_ (s^−1^) (**b**) Catalytic efficiencies, *k*_cat_/*K*_M_ (s^−1^ M^−1^) (**c**) Michaelis constants, *K*_M_ (mM) (d) Dissociation constants, *K*_D_ (μM). Values reported in (**a**–**c**) and (**d**) are derived from the fit to the Michaelis-Menten equation and Stern-Volmer equation, respectively.

In Arr-wd 1–4 proteins, the specific residue X is Met, Gly, His, and Lys, respectively. To evaluate whether the least conserved residue in the M[R|K][D|E]XL motif plays a role during the enzyme catalysis, we prepared three Arr-wd3 variants, H125M, H125G, and H125K (Additional file 1: Table S4); Met, Gly, and Lys are the amino acids that other Arr-wd 1, 2, and 4 proteins natively have at the analogous position, respectively. Although turnover rates (*k*_cat_) (Fig. 6a; Additional file 1: Table S8) and MIC values (Additional file 1: Table S9 and Figure S8) are marginally perturbed by the H125 mutations, the mutants H125M, H125G, and H125K display enhanced *k*_cat_/*K*_M_ for all rifampin analogues. The data demonstrate that this residue is involved in the enzyme activities, and the relatively low activities of Arr-wd3 are at least partially attributed to the steric hindrance with little flexibility of the histidine residue at this position.

It is also supported by the *K*_D_ values of the H125 variants (Fig. 6d; Additional file 1: Table S7). H125G mutation yields the largest decrease in *K*_D_ value, followed by H125M and H125K, for all rifampin analogues, implicating that the size and the flexibility of the side chain is correlated with the degrees of favorable interactions with the rifamycins.

The most dramatic enhancement of the *k*_cat_/*K*_M_ is observed from the reaction with rifaximin. When Arr-wd3 protein is compared with the H125G variant, the *k*_cat_/*K*_M_ is increased by 16-fold, indicating that the removal of the imidazole ring presumably alleviates the steric clash with the bulky and strained 5 and 6-membraned rings fused to the naphthoquinone core of rifaximin. In contrast, all Arr-wd3 mutants exhibit the least degree of enhancement upon the reaction with rifapentine or rifampin. The reactivity pattern with the rifampin analogues are consistent with our observation described above that rifaximin and rifapentine are the least and the most promiscuous substrates, respectively. These data suggest that the combined effects of the chemical structures of the antibiotics and their dynamic interactions with the enzyme active site may determine the enzyme catalysis, and the X residue in M[R|K][D|E]XL motif plays an important role in the reaction.

Even with the low sequence conservation in the X residue of the M[R|K][D|E]XL motif (Fig. 1a), the role in the reactivity and biochemical function was revealed only after the examination of protein structure. Therefore, the current study suggests that integrative analysis of 1-dimensional sequence and 3-dimensional structural information can provide us comprehensive information to elucidate the function of unidentified genes.

## Conclusions

Using metagenomic analysis on the sediment microbiome, we have identified new ADP-ribosyltransferase genes that are involved in the development of antibiotic resistance. These proteins share low sequence similarity with known enzymes, but have highly conserved amino acid residues around the enzyme active site, which catalyzes the transfer of NAD^+^-derived ADP-ribosyl group to the antibiotic rifampin.

To gain a detailed understanding of this process, we carried out extensive analysis of gene sequence and protein structure to locate functionally relevant regions. For experimental validation of our finding, we expressed four representative genes into the proteins: Arr-wd1, Arr-wd2, Arr-wd3, and Arr-wd4. These proteins are kinetically competent in the ADP-ribosylation of rifampin using NAD^+^. Steady-state kinetic analysis and binding assays revealed that the Arr-wd proteins exhibit much tighter binding interaction with rifampin and its analogues than previously reported Arr proteins. The steady-state activities of the Arr-wd proteins are altered depending on the substructures of the rifamycin substrates, and site-directed mutagenesis  studies provided important chemical insights into how the steric interaction between the protein and substrate might affect the enzyme activity.

This work nicely demonstrates the advantage of an integrated bioinformatics approach to discover new enzyme functions from environmental microbiome data. Taking a step further from simple sequence analysis and classification, we have constructed three-dimensional models to identify structural features important for enzyme activity, and validated computational predictions with biochemical experiments. Our studies indicate that chemically guided integrative approach can improve the quality of the metagenomic data and can provide greater knowledge to predict structure-function relationships, thereby facilitating the annotation of protein function with high accuracy. Sequence alignment and site-directed mutagenesis also suggest that even a single amino acid residue with low sequence conservation can alter the *in vitro* and *in vivo* enzymatic activities, which further highlights the importance of identifying key enzyme residues to understand and combat antibiotic resistance.

## Methods

### Data collection

A microbiome sample was collected from the soil sediment (latitude 33°49′65″N longitude 126°96′84″E), and sequenced using Hiseq™ 2000 platform (Illumina, San Diego, USA). The sample was prepared according to the Illumina protocols. For a 350 bp insert size, one microgram of genomic DNA was fragmented using Covaris. The raw sequencing data described in this study is available at European Nucleotide Archive (ENA) with the accession number PRJEB25358. The 151 bp paired-end sequencing reads were assembled by using MegaHit^48^ with the default option. The genes predicted by FragGeneScan^49^ were analyzed to find the rifampin resistance functions. Seven *arr* genes (i.e. *arr*-1, *arr*-2, *arr*-3, *arr*-4, *arr*-5, *arr*-7, and arr-8) were obtained from the comprehensive antibiotic resistance database (CARD)^41,42^. These genes were used as query to search homologous genes in the microbiome sample by blastp with the threshold of 50% sequence similarity and e-value < 1.0e-10. As a result, 45 homologous genes were obtained (Fig. 1a; Additional file 1: Table S1).

### Phylogenetic analysis

In order to delineate the phylogenetic relationship between the putative *arr* genes and the annotated genes from the sequenced genomes, a phylogenetic tree was constructed (Fig. 3). The known *arr* genes were obtained from the CARD database and NCBI repository. In order to find homologous *arr* genes in the microbial genomes, 45 *arr* genes were searched against a set of 29,574,723 protein sequences of 8,369 bacterial genomes (downloaded from NCBI ftp server on 12/05/2017). In the phylogenetic tree, we included 45 *arr-wd* genes, 7 known *arr* genes from CARD database, 8 *arr* genes from the previous study^13^, and 37 putative *arr* genes that were searched from the microbial genomes. For each *arr* gene, five most homologous sequences (evalue < 1.0e-10) were obtained, and a set of non-redundant sequences were retained to build the phylogenetic tree. Neighbor-joining method was applied to build the tree with the bootstrapping value of 1000. MUSCLE^50^ and MEGA^51^ were used for multiple sequence alignment and phylogenetic analysis, respectively.

### Integrated analysis of protein structure and sequence

Our integrated analysis of ADP-ribosyltransferase exploits rifampin and NAD^+^ binding sites. For this purpose, both structure-guided and sequence-based approaches were taken. The structure-guided conservation analysis reveals the amino acids that are sterically and/or spatially conserved and physico-chemically similar, but relatively low in sequence conservations. The analysis consists of three tasks: (1) multiple sequence alignment (MSA) of the reference *arr* genes; (2) ligand-protein complex binding analysis between rifampin and Arr*-ms*; (3) mapping conserved residues from MSA with featured amino acid residues interacting with rifampin. A previously reported X-ray structure of Arr*-ms* complexed with rifampin (PDB ID: 2HW2) was used to find amino acid residues involved in the ADP-ribosylation activity. Both intermolecular (i.e. between rifampin and Arr-*ms*), and intramolecular interactions of Arr-*ms* are considered in the rifampin binding sites. The molecular interactions are determined from the distances between the atoms that participate in hydrogen bonding or hydrophobic interactions. The results were incorporated with sequence conservation data obtained from metagenome analysis.

No crystal structure is currently available for Arr proteins complexed with NAD^+^. We thus performed Autodock^52^ simulation to visualize possible orientations of NAD^+^ when bound to the protein. The size of grid box was set to 22 × 24 × 28 Å^3^. Structural models of Arr-wd were generated from the Swiss modeling^53^ to simulate their possible three-dimensional structures (Additional file 1: Figure S2). PyMol^54^ was used to visualize the X-ray crystal structure of Arr-*ms* (Figs 1–2; Additional file 1: Figures S1–S2 and S5). Discovery Studio Visualizer^55^ was used to generate the 2D diagram of molecular interactions between rifampin and Arr-*ms*. The sequence alignment figure was generated by JalView^56^.

### Protein expression and purification

Among 45 homologous genes from the metagenomic analysis, four genes denominated as arr-wd1, arr-wd2, arr-wd3, and arr-wd4 were selected as the targets for biochemical characterizations (Fig. 1a; Additional file 1: Table S3). The codons of the DNA fragments were optimized for *E. coli*. expression prior to the gene synthesis (General Biosystems), and were inserted into pET22b(+)/amp^R^ vector using NdeI and XhoI cut-sites. Each plasmid was transformed to either DH5α or BL21(DE3) competent cells for sequencing or protein expression, respectively. All proteins were expressed with a His-tag at the C-terminus, and Arr-wd3 without the His-tag (Arr-wd3 + Δ6His) was additionally prepared to ensure that the presence of His-tag yields no significant perturbation in their activities. Single variants such as N34H, H125M, H125G, and H125K were prepared by site-directed mutagenesis. The PCR was carried out with KOD DNA polymerase (Toyobo) and primers (Additional file 1: Table S4). After PCR, the reaction products were digested with DpnI and transformed to DH5α competent cells. The plasmids were extracted by using miniprep kits, and sequenced at Macrogen, Inc.

For protein overexpression, a single colony of BL21(DE3) was picked and inoculated in 10 mL autoclaved LB media containing 100 mg/L ampicillin. The culture was grown in 200 rpm orbital shaker at 37 °C overnight, and inoculated in 1 L autoclaved LB media containing 100 mg/L ampicillin. When the optical density at 600 nm (OD_600_) reached ca 0.7, 1 mM isopropyl β-D-1-thiogalactopyranoside (IPTG) at the final concentration was added. After 3 h of induction at 37 °C, the cells were harvested by centrifugation at 5000 rpm (4715 × g) for 15 min at 4 °C. The cell pastes were frozen in liquid nitrogen and stored at −80 °C for further usage.

The protein purification was initiated by thawing the cell pastes in lysate buffer (20 mM sodium phosphate buffer, pH 7.4 with 500 mM NaCl). The cells were lysed by sonication for 15 min in an ice bath (on/off = 3 s each). The lysates were centrifuged at 13000 rpm (18800 × g) for 30 min at 4 °C, and the supernatants were loaded to Ni affinity column (HisTrap HP column, GE Healthcare Life Sciences). The column was pre-equilibrated with the lysate buffer at 4 °C using ÄKTA Protein Purification Systems. After loading the cell lysates, elution buffer (20 mM sodium phosphate buffer, pH 7.4 with 500 mM NaCl and 500 mM imidazole) was applied in a linear gradient (5–50%) (Additional file 1: Figure S3). All proteins were eluted when ca 100 mM imidazole was applied. The protein expressed without a His-tag (Arr-wd3 + Δ6His) was purified by Q anion exchange chromatography by varying the concentration of NaCl (0–1 M) in 20 mM sodium phosphate buffer, pH 8.0 at 4 °C. Relatively pure fractions (ca 80–90%) were concentrated using Amicon stirred cells (EMD Millipore) or centrifugal concentrating devices with 10 *k*Da cutoff membrane filters; the protein was further purified by size exclusion chromatography (S75 column), eluted with 20 mM Tris/HCl buffer, pH 7.0 with 150 mM NaCl. The purity of the protein was determined by sodium dodecyl sulfate-polyacrylamide gel electrophoresis (SDS-PAGE). The mass of the protein was confirmed by matrix-assisted laser desorption/ionization-time-of-flight mass spectrometer (MALDI-TOF; Bruker Ultraflextreme TOF/TOF) with sinapinic acid as matrix solution. The protein was concentrated up to ca 0.5–5 mM and stored at −80 °C until further usage. The protein concentration was determined by UV–vis spectrophotometer (Agilent Cary 8454). The extinction coefficients at 280 nm were estimated from the sequence (Additional file 1: Table S3)^57^.

### *In vitro* activity assay

The assays were carried out in dark due to the light-sensitivity of rifampin and its analogues. Rifampin was dissolved in methanol, and diluted with the buffer used for the assay (Fig. 4). Initially, we followed the previously reported procedure (Additional file 1: Figure S4a)^13^. In short, 10 μM protein in 100 μL 50 mM 4-(2-hydroxyethyl)-1-piperazineethanesulfonic acid (HEPES) buffer, pH 7.5 was mixed with various concentrations of the substrates, NAD^+^ and rifampin. The solution was left for 5 min at 22 °C, and the reaction was quenched by the addition of 100 μL methanol. The reaction mixture was analyzed by HPLC (Agilent 1260 Infinity II, C18 column with 2.7 μm, 120 Å, 4.6 × 100 mm). We later modified the quenching method by increasing the volume of methanol to 400 μL because 100 μL methanol was not sufficient to completely quench the reaction (Additional file 1: Figure S4b). In addition, we modified the HPLC method to accurately detect the formation of nicotinamide from the reaction mixture as follows: 100% H_2_O and 0.05% trifluoroacetic acid (TFA) for 3.5 min, followed by a linear gradient to 90% CH_3_CN with 0.1% TFA over 9.5 min (Fig. 4a). The ADP-ribosylated products were confirmed by electrospray ionization mass spectrometry (ESI-MS; Thermo Finnigan LTQ) (Additional file 1: Table S5).

Steady-state kinetic parameters of Arr-wd enzymes were obtained by varying the concentrations of one of the substrates, either NAD^+^ or rifampin, when the other was fixed to be 1 mM. The product yields were measured by detecting the concentrations of nicotinamide by HPLC. The standard curve of nicotinamide was measured independently (Additional file 1: Figure S4c). The kinetic parameters, *k*_cat_, *K*_M_, and *k*_cat_/*K*_M_, were determined from non-linear iteration curve fits to the Michaelis-Menten equation (Figs 4 and 5; Additional file 1: Table S6 and Figure S4). Activities of Arr-wd3 variants were also measured by the same procedure as described above (Fig. 6; Additional file 1: Table S8).

*In vitro* activity assay was also carried out with NADP^+^, NADH, or NADPH, instead of NAD^+^, and with rifampin analogues, such as rifapentine, rifaximin, and rifabutin (Fig. 5; Additional file 1: Figure S6). Representative HPLC traces, Michaelis-Menten fits, and kinetic parameters are included in the Additional file 1: Table S6 and Figure S7. Rifampin analogues and the corresponding ADP-ribosylated products were eluted at 10.6 min and 9.1 min for rifapentine, 10.3 min and 8.6 min for rifaximin, and 9.9 min and 8.4 min for rifabutin, respectively. The reaction products were further analyzed by ESI-MS (Additional file 1: Table S5).

### Determination of binding constants by intrinsic fluorescence changes

Intrinsic tryptophan fluorescence changes were monitored with microplate reader (Biotek Synergy H1m) at 22–25 °C. Arr-wd1-3 and Arr-wd4 proteins possess two and four tryptophan residues, respectively. Sequence alignment together with structure homology modeling suggests that two tryptophan residues are located at similar positions in Arr-wd1–4 (Additional file 1: Figure S5). Conformational changes induced by the addition of substrates were monitored by using 295 nm excitation to avoid any possible interference from tyrosine and phenylalanine residues. The fluorescence emission was observed at 325–450 nm, and the fluorescence emission at 325 nm was used to obtain the dissociation constants (*K*_*D*_). Because rifamycins exhibits strong absorption at both excitation and emission wavelengths (Additional File 1: Figure S5b), the concentrations of rifamycins were kept at 1–4 μM so that the absorption at both excitation and emission wavelengths remains less than 0.05, thereby minimizing the inner filter effect^58^. The plot of F_0_/F versus the concentrations of rifamycins yielded a linear correlation (Additional File 1: Figure S5c), which is fitted to the Stern-Volmer equation (Additional File 1: Figure S5d) to determine the dissociation constants (*K*_D_) for rifampin and its derivatives (Figs 4e, 5d, 6d, and Additional file 1: Table S7). The protein samples (5–20 μM) were prepared in 100 μL of 50 mM HEPES buffer, pH 7.5.

### Determination of MIC values

Minimal inhibitory concentration(MIC) values for BL21(DE3) cells containing arr-wd genes were measured by the procedures reported previously^13^. In short, the plasmids containing arr-wd genes were transformed into BL21(DE3) cells, and were grown on the LB/agar plate containing 100 mg/L ampicillin overnight at 37 °C. The cells were mixed with 0.85% saline to adjust the OD_625_ to be 0.1. The aliquots of 100 μL cell culture in saline solution were mixed with 18.9 mL Mueller Hinton broth media and 0–512 μg/mL rifamycin dissolved in methanol. Either 0 or 100 μM IPTG at final concentration was added to the solution. After the solutions were incubated at 37 °C overnight, optical cell density (OD_625_) was measured with the microplate reader to determine the minimum inhibitory concentrations of the antibiotics (Fig. 3f; Additional file 1: Table S9 and Figure S8).

## Electronic supplementary material

Additional File 1

## Data Availability

The raw sequencing data described in this study is available at European Nucleotide Archive (ENA) with the accession number PRJEB25358.
