# Supplementary material for: Integrative metagenomic and biochemical studies on rifamycin ADP-ribosyltransferases discovered in the sediment microbiome
Source: Sci Rep. 2018 Aug 14;8:12143. doi: 10.1038/s41598-018-30547-x (PMC6092378; doi:10.1038/s41598-018-30547-x)
Supplement: Supplementary file 1 — Additional File 1 [file 41598_2018_30547_MOESM1_ESM.pdf]

Additional file 1: Scheme S1, Table S1–S9, and Figures S1–S8

Integrative metagenomic and biochemical studies on rifamycin ADP-ribosyltransferases discovered in the sediment microbiome

Jae Hong Shin[1,†], Hyunuk Eom[2,†], Woon Ju Song[2,*], Mina Rho[1,3,*]

[1]Department of Computer Science and Engineering, Hanyang University, Seoul, Korea

[2]Department of Chemistry, Seoul National University, 1 Gwanak-ro, Gwanak-gu, Seoul, 08826, Korea

[3]Department of Biomedical Informatics, Hanyang University, Seoul, Korea

[†]These authors contributed equally to this work.

*Corresponding authors: woonjusong@snu.ac.kr, minarho@hanyang.ac.kr

Scheme S1. Reaction of mono-ADP-ribosyltransferase (a) ADP-ribosylation of rifampin. (b) Uncoupled reaction of NAD^+ consumption.

(a)

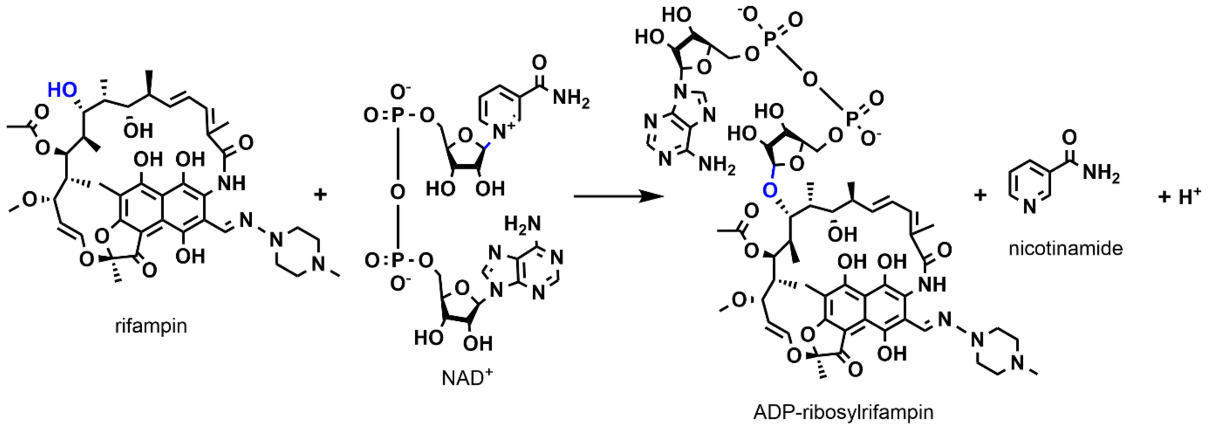

(b)

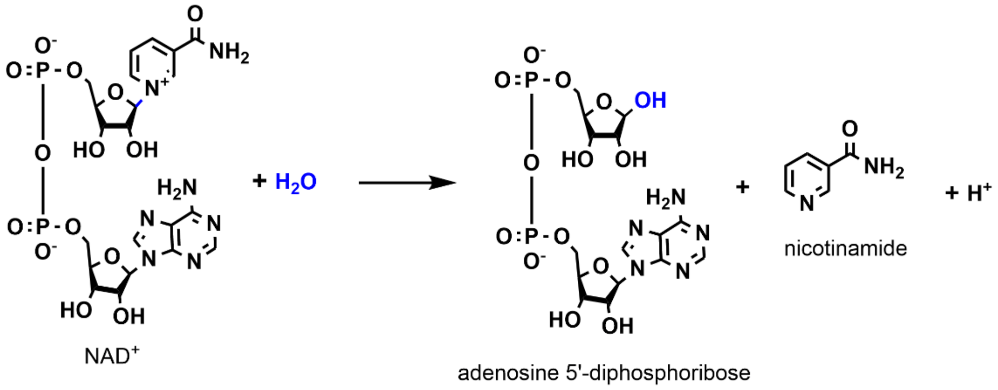

Table S1. A total of 45 rifamycin ADP-ribosyl transferase (arr) homologous genes obtained from the sediment microbiome

Table S2. Protein sequence similarity between 45 newly identified *arr* genes and the genes in the complete genomes

Table S3. The list of nucleotide and amino acid sequences of four representative Arr-wd proteins involved in rifamycin-resistance

Table S4. Primer sequences used for the current work.

Table S5. ADP-ribosylated product analysis by ESI-MS.

Table S6. Steady-state kinetic parameters of Arr-wd proteins in ADP-ribosylation of rifampin and its analogues.

Table S7. Substrate dissociation constants (K_D) measured from the intrinsic fluorescence quenching.

Table S8. Steady-state kinetic parameters of Arr-wd3 variants in ADP-ribosylation of rifampin analogues.

Table S9. Determination of minimum inhibitory concentration (MIC) of rifampin and its analogues.

Table S1. A total of 45 rifamycin ADP-ribosyl transferase (arr) homologous genes obtained from the sediment microbiome.

>W2_1488688
DGIVGPFFHGTHADLSPGDLIAPGYPSHYGARKLASWVYMTRIVAGAVLAAEIAAGEGRARVYLVEPTGPFEDDPNVTDKKFP
GNPTQSYRTKDPLRVLEEVLDWAPTPAERLAQLRDFVERTRREGIEAID
>W2_1852679
EFSPNNFVVKLCIQGMALEDEGKPEEADKIFLQAWDEAANDFEKFTAAYYVARHQNNSADKLKWLETVLEFGLKINDDTVKSA
FPALYFNIAECYEDLGDAENAKKNYELADSFKHKPSDKGPFYHGTRADLQVGDLLTAGGKSNYKAELTMNHIYFTALVNGAGLA
AALVGGDGRERVYIVEPTGSFENDPNVTDKKFPGNPTRSYRSEAPLEIVGEVTDWVKITPEELQKWREKLANNKGEIIN
>W2_2666971
EFSPNNNVVKLCIQGMALEDEGNPEEASKLFLQAWNEAANDFEKFTAGYYVARHQNNIADKLKWLETVLEFGLKINDDTVKSA
FPGLYLNIAECYEDLGDVENAKKNYELADSFKHKPSDKGPFYHGTRADLQVGDLLTAGGKSNYKAELTMNHIYFTALINGAGLA
AALASGDGRERVYIVEPTGSFENDPNVTDKKFPGNPTRSYRSQAPLKIVGEATDWARITPEELQKWRKKLANSKGEIIN
>W2_399810
DENKEKNNLNRQGATPFAQTYFHGTKANLKIGDLIEAGFNSNYGVRKNAKYIFLSATLDAAIWGAELAFGEGRERIYLVEPTGQI
EDDPDLTDKKFPGNPSKSYRSTEPFRVVGEVGSWQRHPAEQVNTMKAHLEKLKAQGINSLNDE
>W2_931432
EFNPNNNIVKLCLHGMSLEDGGKPEEASEIFLQAWNEAENDFEKYLAAYYAARHQKNAADKLQWLETALEFASKVNTDSAKS
AFPSLYANIAKCYEDLGDSENAKKHNDLADSFKNEPSDKGPFYHGTRADLQVGDMLTAGGKSNYKAELVMNHIYFTAIANGA
GLAAALANGDGRERVYIVEPTGSFENDPNVTDKKFPGNPTRSYRSQSPLKIVGELTDWARITPEQLREWREKLANSKGEIIN
>W2_1076727
EPEKSVPTSIQSPFAQTYFHGTKADLKIGDYIEAGYHSNYGQRKNARYIFLTATLDAAIWGAELAFGEGRERIYLVEPTGPIEDDP
DLTDKKLPGNPTKSYRSTHPFKVVGEVTLWQGHPVAQVKAMKEGLAKLKEQGINSLNDE
>W2_1252150
EENNTTTQTMQRIFVQTYFHGTKADLKMGDIIETGITSNYGQRSNAKYIYLTATIDAAIWGAELAFGDGRERIYLVEPTGPIEDD
PNLTDKKFPGNPTKSYRSKHSFKVVGEVTVWQRHSAEQVKAMKDHLQKLKDEGIEAIED
>W2_493312
TDRQSPCEGVTYYHGTRADLKPGELLRPGYGSNYTERASPWVYFSATLEAATWGAELARGDGPGRIYVVEPTGAFVDDPNLTD
KRFPGNPTKSYRSQEPLRVVAEHVGWQGHTPEAVAAMKAAISGLEPIDD
>W2_1280285_wd4
EFSPNNNVVKLCLQGMELEEKGKPEEASEIFLQAWNEAADDFEKFTAGYYVARHQNDTADKLKWLETVLEFGLKINDDTVKSA
FPALYQNLAKCFEDLGDARNAKKNYELANSFKKKPSDKGPFYHGTRADLQIGDLLTAGGKSNYKAELVMNHIYFTALVNGAGL
AAALANGDGRERVYIVEPTGSFENDPNVTDKKFPGNPTRSYRSQAPLKIVGEVTDWVRITPEELQKWREKLANSKGEIIN
>W2_1524164
TDQHPNFIQSYFHGTKSNLSVGDLIVPGKDANYGERKAKFVYASSNLNVAIWGAELAVGDGPGRIYVVEPMGTVENDPNLTD
KKFPGNPTNSYRAQNGFKVVAEVKDWQGHPPDEIKAAREKVDLDLKNGAPIIED
>W2_2157429
NDKKDVLDNGPFFHGTKAELKIGDLLEPQHLSNYQNKKSNYIYFTATLNAAKWGAELATSKSKERIYIVEPLGEFENDPNLTDKR
FPGNPTRSYRSKSPLKIIAELGSWERHSDEEINHMISSLKKLSDEGKNVIYD
>W2_1786964
VLVYLQHYFTPLQKKGFCEYIKLPTIKMEQYYHGTKAQLNIGELIEPGFRSNYGIRKKANFVYLTATLEAATWGAELASGDGPGRI
YIVEPTGSIEDDPNLTDKKFPGNPTKSFRSRHALKIVGEVEQWEGHTNEQLDNMRAHLERLKASGIEAIEE
>W2_1931402
NSDESRATPFAQTYFHGTRADLRVGERIEVGFASNFGQRTNARYIFLTATLDAAIWGAELALGDGRERIYLVEATGPVEDDPDLT
DRKFPGNPTMSYRSTHPFRVVGEVGGWQGHPSEQVGAMREALDKLNEQGVTSLNDQA
>W2_611506
PDRHPNFIQSYFHGTKANLRIGDLIVPGNSANYGERMAKYVYASSNLNVAIWASELAVGDGPGRIYVVEPIGAIENDPNLTDKK
YPGNPTNSFRAREGFKVVAEVKEWLGHSPEEIKAAREKVDLALKNGAPIIED
>W2_2027090
ENTTIDLSQIFYHGTKAELKTGDFIEPGYNSNYGKKKKAKYIYLTATLDAAIWGAELAAGEGRQRIYIVEPTGTIEDDPNLTDKKFP
GNPTKSYRSMHPLKVIGEVSEWTGHSPEQLKAMKDHLAELERMGIEAIED
>W2_1316525
SSDQPRATPFAQTFFHGTKADLGLGDLIEVGFASNFGRRTNAKYIFLTSTLDAAIWGAELALGDGRERIYLVEATGPVEDDPDLT
DRKFPGNPTMSYRSTHPFRVVGEVRVWQGHPPEQVSAMRDGLAKLKEQGVRSLNE
>W2_1614168

EFDPNNNVVKLCIQGMALEEKDKPEEAGKLFLQAWNEAGDDFEKFLAAHFVARHQKNVPDKLKWLETALQFALKINNDSVKS
AFPSLYSNIARCYEEMGDAANAKKNYESAVSFKDNPSDKGPFYHGTRADLQIGELLTAGGRSNYKPELTMNHIYFTALVNGAGL
AAALAKGDGRERVYIVEPTGSFENDPNVSDKKFPGNPTRSYRSQSPLKIVGEVTDWVRQTPGQIQKWREKLADNKGKIIN
>W2_705194
NQPTESDHHQGATPFVQTYFHGTKAALKIGALIEPGYHSNYGQHKTATYIFLTATLDAAVWGAELSLGEGPERIYLVEPTGPIED
DPDLTDKKFSGNPTKSYRSLYPFRVVGEVASWQGHPETQVNMMKDHLAKLKEKGINSLND
>W2_691914
PDVPAASVAGGTRDAGRRHGPGASLNRTNVASDVRRRAVRTHLLERDMDGDGSDPGPFFHGTKADLQVGDLLTAGFRSNYR
PDVLMNHVYFTALVGTAALAAALAAGDGRERVYVVAPTGPFENDPNVTDKKFPGNPTRSYRSQSPLTVVGEVADWVRLTPEE
RAAWHERLAAITGEIIN
>W2_1580944
EFNPNNNIVKLCLQGLDLEEKGKLKEASGIFLRAWNEAADDFEKFIAAHYVTRYQKDVSDKLKWIETSLRLALEMDSDYAKGVLS
SLYLKKARCFEESGDAGNAKKNYELAESFKHKLSDNGPFYHGTRADLQVGDLLTAGFRSNYHSEVVMNHIYFTAIVNGAELAAE
LAKGDGRERVYIVEPTGSFENDPNVTDKKFPGNPTRSYRTREPLKIVGEVTDWVRLTSEQLQTWREKLANIEKAEIIN
>W2_456471
TELDSGPFYHGTKADLQVGDMLTAGFQSNYDDRVVMNHIYFTALPKGAGLAAEMAKGDGKPRVYVVEPTGEFENDPNVTDK
KFPGNPTRSYRSKHPLRIIGSLDSWEPFDPEFVQNLRSRVKAGLGEIIN
>W2_1294492
EFTPTNPIIRRCLQGMALEAAGKPEEAGQLFLQAWNEATHDLEKFLAAYYVARHQKNDSDRLHWLETALACALKTNTESVKSA
YSMLYLNIAKCHEALGNSDEAKKNYALAASFQGTPADQGPFYHGTKADLEVGDVLTAGGTSNYQPDLKMNHIYFTALAHGAG
LAASLAKGEGRERVYIVEPTGDFENDPNVTDKKFPGNLTRSYRSRVPLKIVGELTEWARQTPEELQKFREKLATNQGGIIN
>W2_2321962
NPNTTEKNNPGATPFAQTYFHGTKADLKLGDLVEPGFNSNYGEQKNAKYIFLTATLDAATWGAELAFGEGRERIYLVEPTGPIE
DDPDLTDKKFRGNPTKSYRSRHPFKVVGEVTVWQGHPAQQVQLMKDHLQQLHQQGIRSLNDE
>W2_1976254
DQTPITNNASATPFAQTFFHGTKAALNIGDLLQVGFNSNYAQYGQLNHLYMTATLDAAIWGAELASGEGKERIYLVEPTGSIED
DPNLTNKKFPGNPTMAYRTRQPLKVVGEVATWQGHTKEQVKAMKDGLLKLREEGLDGIEN
>W2_2509215
QNPNKNLMQGNKNNQQLDNAGPTPFFQTFFHGTRADVKIGGEISVGYYSNYGDRKNANYVFLTATLDAAIWGAELAVGNA
GERIYLVEPTGLIEDDPDLTDKKFPGNPTKSYRSKHPLKVVGEVATWQSHPPEQIEAMKEGLERLKAKGVTSLNE
>W2_436021
TYAKDATPPFYHGTRADLHVGDLLAPGFASNYQEQKLSWIYFSGTLDAAIWGCELAKGDGPERIYVVEPTGAFEDDPNLTDKRF
PGNPTRSYRSRAPLRIVGEVTEWEPHPPERLEEMKDALARMEAEGDMKIIDWTTVDEGALNKCADDEWLYIEAWDES
>W2_982741
EQNQSNTNACPKPFAQTYFHGTKAALEIGDLLQVGFNSNYKESDHLNHVYMTATLDAAIWGAELASGDGRERIYLVEPTGTIE
DDPNLTNKKFPGNPTMAYRSQHPLRVVGEVTLWQGHAPEQIKAMKDGLAKLREQGLDTIEN
>W2_2284918
SSDRGTNYYMTFSTNNHIVKLCLQGMALEENGKPGEASRLFLQAWNEATNDHEKFFAAYYLAQRQSNVADKLKWLETALQF
ALHINDDSVKSAFHSLYSNIAKCYEELGDVDNAKKNHDKASSFKDNPSDKGPFYHGTKVDLQVGDLLTAGFGSNYQSDLVMNH
IYFTALVDGAGLAAALAKGDGRERVYIVEPTGSFENDPNVTDKKFPGNPTRSYRTQSPLKIIGEVPDWIRTPTEEVRKWREKLAN
NKGEIVN
>W2_928841
EFDPNNNVVKLCIQGMNMEEKDRPEEASGLFLEAWNEAGSDFEKYLAAYYVARHQDDVHDRLKWLETSLKFALKINDVTVKS
AFPSLYSKIADCCEDLNDSDNAKKNRELANSFTENLPDSGPFYHGTRADLKVGDLLTAGFGSNYKSDLTMNHIYFTALVNGAGL
AAALAKGDGPERVYIVEPTGRFENDPNVTDKKFPGNPTRSYRSQSPLKIVGEVTNWARLTPEQLQQWREKLANTKGEIIN
>W2_1056966
ASSDDPTPTIYYHGTKADLAPGDQIAPGNRSNYGARKPATFVYLTATLDAATWGAELAVGDGRGRIYTVEPTGPIEDDPNLTDK
KYPGNLTRSYRTRDPLRVTGEVADWQGHPPEALQAMRDHLEHLRALGVEAIED
>W2_759404
NPTSPSPFSQTFFHGTKADLKMGDLIQPGYQSNYTAHEQLNHVYFTATLDAAIWGAELANGEGRQRIYLVEPTGEMEDDPNV
TNKRFPGNPTRSYRSIQPVRVVGEVAVWQGHPPEQVNGMKEALAKLREEGRDTIEN
>W2_505284
IEPGFSSNYGARKKAVWVYLTATLDAATWGAELALGEGRGRIYTVEPTGPIEDDPNLTDKKFPGNPTRSYRSRHPLRVTGEITD
WEGHPPEAIAGMKAHLERLKELGIEAIED
>W2_2302663
TASDDGLPQQYYHGTKADLKPGDLIAPGFSSNYGERKKAAYVYLTATLDAATWGAELAVGEGRGRIYVVEPTGPIQDDPNLTD

KRYPGNPTKSYRTRHPLRVTGEVTDWQGHPPEQLEAMRAHLEQLERLGIEAIED
>W2_1051501
TATNDSNSETFYHGTRADLKPGDLIEPGYSSNYGARKKAAFVYLTATLDAATWGAELAVGEGRGRIYIVEPTGPIEDDPNLTDKK
FPGNPTKSYRTRDPLRVTGEVLDWTGHSPEQLQTMKDHLERLKEMGIEAIED
>W2_1844246
ETNRNENQSQARASDLSREIFYHGTKADLKPGDLIEPGFYSNYGKRKKANYVYLTATLDAAVWGAELALGEGPGRIYIVEPTGP
MEDDPNLTDKKYPGNPTKSYRSQSPLRITGEITDWQGHTPEQLNTMKEHLERLKQLGIEAIED
>W2_1355083
ETNKSEDKSIESAYDLSSQAFYHGTKANLKQGDLIEPGFNSNYGQRKKAKYIYLTATLDAAIWGAELAVGEGDGRIYIIEPTGPFE
DDPNLTDKKFPGNPTKSYRSLHPLHVIGEVTDWQGHSPEQLNAMKEHLERLKQLGIEAIEE
>W2_1008891
SDKPMAAIDDLSSQRFYHGTKADLKPGDLIGPGYNSNYGKRKKAAYVYLTATLDAAAWGAELAVGEGRGRIYIVEPTGPIEDDP
NLTDKKYPGNPTKSYRSRDPLRVTGEVTDWQGHSPEQLRAMKDHLDHLRQLGVEAIED
>W2_1930875_wd3
DKTADDARTAQPFYHGTKADLKPGDLIAPGYASNYGARKKAAFVYLTATLDAATWGAELAAGEGRGRIYLVEPTGPIEDDPNLT
DKRFPGNPTKSYRSRDPLRVTGEVTDWQGHPHEQLQAMREHLERLRAQGIEAIED
>W2_498816
THDDLTSQQFYHGTKADLKPGDLIEPGYNSNYGKRKKASYVYLTATLDAATWGAELAFGEGPGRIYTVEPTGPIEDDPNLTDKK
YPGNPTKSYRTRSPLRVTGEIVDWQGHPPEQLKAMKDHLAQLERLGIEAIED
>W2_1851401
TTDDLSPPRYYHGTKADLRPGDLIAAGYRSNYGERRKAAYVYLTATLDAATWGAELALGEGPGRIYVVEPTGEIEDDPNLTDKKY
PGNPTKSYRTRDPLRVTGEVADWPGHPPEQLQAMRDHLDRLRESGIEAIED
>W2_1182683
RSEPMGAIDDLDSQRFYHGTKADLKPGEMIGPGYNSNYGKRKKAAYVYLTATLDAAVWGAELAVGEGPGRIYTVEPTGPIEDD
PNLTDKKYPGNPTKSYRTRDPLRVTGEITDWQGHSPEQLKAMKDHLEQLRRLGIEAIED
>W2_317439_wd1
SDTMPPEGAPRLPPFYHGTKADLKPGDLIAPGFRSNFGSGRAAAYVYLTATLDAAVWGAELALGDGRERIYVVEPTGPIEDDPN
LTNAKYPGNPTQSYRTRDPLRVSGEVTSWQGHTPEQLREMREMVARMRRRGIEAIED
>W2_770591
MTSENARSERFYHGTKADLKPGDLIAPGYTSNFGKRKKAAYVYLTATLDAATWGAELALGEGAGRIYIVEPTGPIEDDPNLTDKK
YPGNPTKSYRSRAPLRVTGEITDWQGHSPEALKAMKDHLAQLRRLGIEAIED
>W2_1428970_wd2
QTNKTENKSNEPSQSPFVQTFFHGTKADLKIGDFIEVGLNSNYGQRKNAKYIYLTATLDAAIWGAELALGEGRERIYLVEPTGPIE
DDPNLTDKKFPGNPTKSYRSKHPFKVVGEITIWQGHSPEQVKAMNEGLAKLKEQGIEAIED
>W2_921462
TDGPKPFEVHESGALLHGTKADLAIGDMLVPGYLANYGEERRANHVYVTETLDAAAWAAELAVGPGRGRIYFVEPTGPLEDDP
NLTDKKFPGNPTRSYRTRAPVKVVGELVDWVGHSEEQQQAMRDGLADLERRGLAIVEDD

Table S2. Protein sequence similarity between 45 newly identified *arr* genes and the genes in the complete genomes

Arr gene name	Accession number	Species name	Sequence similarity (%)
W2_1488688	WP_069949724.1	*Arthrobacter sp. U41*	57.81
W2_1852679	WP_084490216.1	*Niabella ginsenosidivorans*	74.39
W2_2666971	WP_084490216.1	*Niabella ginsenosidivorans*	76.83
W2_399810	WP_077133846.1	*Spirosoma montaniterrae*	80.14
W2_931432	WP_084490216.1	*Niabella ginsenosidivorans*	75.20
W2_1076727	WP_053195305.1	*Herbaspirillum hiltneri*	82.35
W2_1252150	WP_096357565.1	*Mucilaginibacter gotjawali*	76.22
W2_493312	WP_015132086.1	*Calothrix sp. PCC 7507*	66.93
wd4	WP_084490216.1	*Niabella ginsenosidivorans*	75.61
W2_1524164	WP_061991201.1	*Flammeovirgaceae bacterium 311*	56.59
W2_2157429	WP_013239403.1	*Clostridium ljungdahlii*	98.54
W2_1786964	WP_048919688.1	*Rufibacter sp. DG31D*	71.97
W2_1931402	WP_077133846.1	*Spirosoma montaniterrae*	73.79
W2_611506	WP_061991201.1	*Flammeovirgaceae bacterium 311*	57.36
W2_2027090	WP_011585690.1	*Cytophaga hutchinsonii*	77.78
W2_1316525	WP_077202317.1	*Vibrio*	78.83
W2_1614168	WP_084490216.1	*Niabella ginsenosidivorans*	78.46
W2_705194	WP_077133846.1	*Spirosoma montaniterrae*	74.45
W2_691914	WP_066405829.1	*Flavisolibacter tropicus*	81.89
W2_1580944	WP_084490216.1	*Niabella ginsenosidivorans*	71.66
W2_456471	WP_069243308.1	*Burkholderia latens*	81.25
W2_1294492	WP_084490216.1	*Niabella ginsenosidivorans*	67.07
W2_2321962	WP_077133846.1	*Spirosoma montaniterrae*	77.24
W2_1976254	WP_080238844.1	*Spirosoma rigui*	78.03
W2_2509215	WP_013930076.1	*Runella slithyformis*	66.89
W2_436021	WP_075153047.1	*Sphingomonas koreensis*	70.77
W2_982741	WP_080238844.1	*Spirosoma rigui*	79.70
W2_2284918	WP_084490216.1	*Niabella ginsenosidivorans*	72.06
W2_928841	WP_084490216.1	*Niabella ginsenosidivorans*	74.80
W2_1056966	WP_015132086.1	*Calothrix sp. PCC 7507*	73.72
W2_759404	WP_080238844.1	*Spirosoma rigui*	71.94
W2_505284	WP_015175623.1	*Oscillatoria nigro-viridis*	81.42
W2_2302663	WP_015171984.1	*Geitlerinema sp. PCC 7407*	80.88
W2_1051501	WP_015171984.1	*Geitlerinema sp. PCC 7407*	81.62
W2_1844246	WP_015175623.1	*Oscillatoria nigro-viridis*	82.27
W2_1355083	WP_015175623.1	*Oscillatoria nigro-viridis*	85.62
W2_1008891	WP_015132086.1	*Calothrix sp. PCC 7507*	84.29
Wd3	WP_015171984.1	*Geitlerinema sp. PCC 7407*	80.88
W2_498816	WP_015171984.1	*Geitlerinema sp. PCC 7407*	85.07
W2_1851401	WP_015171984.1	*Geitlerinema sp. PCC 7407*	82.84
W2_1182683	WP_015171984.1	*Geitlerinema sp. PCC 7407*	84.78
Wd1	WP_015175623.1	*Oscillatoria nigro-viridis*	74.80
W2_770591	WP_015132086.1	*Calothrix sp. PCC 7507*	84.56
Wd2	WP_096357565.1	*Mucilaginibacter gotjawali*	78.72
W2_921462	WP_025433386.1	*Rhodococcus*	81.02

Table S3. The list of nucleotide and amino acid sequences of four representative Arr-wd proteins involved in rifamycin-resistance

	nucleotide and amino acid sequence
arr-wd1	AGCGATACGATGCCGCCCGAGGGCGCCCCGCGCTTGCCGCCGTTCTACCACGGCACAAAGGCCGACCTG<br>AAGCCGGGAGACCTCATCGCGCCCGGCTTCCGCTCCAACTTCGGCAGCGGACGCGCGGCCGCCTACGTC<br>TACCTGACCGCTACGCTCGACGCGGCCGTCTGGGGCGCCGAACTAGCCCTGGGGGACGGCCGCGAGCG<br>CATCTACGTCGTGGAGCCGACCGGGCCTATCGAGGACGACCCTAACCTGACCAACGCCAAGTATCCCGGC<br>AACCCGACGCAGTCGTACCGCACGCGCGACCCGCTCCGGGTCTCCGGCGAGGTGACTTCGTGGCAGGG<br>GCACACGCCCGAGCAGCTTCGGGAGATGCGGGAGATGGTCGCGCGGATGCGGCGGCGCGGCATCGAG<br>GCCATCGAGGAC
Arr-wd1	SDTMPPEGAPRLPPFYHGTKADLKPGDLIAPGFRSNFGSGRAAAYVYLTATLDAAVWGAELALGDGRERIYV<br>VEPTGPIEDDPNLTNAKYPGNPTQSYRTRDPLRVSGEVTSWQGHTPEQLREMREMVARMRRRGIEAIEDLE<br>HHHHHH (149 aa, 16.6 *k*Da, ε_{280} = 19940 $cm^{-1}M^{-1}$)
arr-wd2	CAAACTAACAAAACTGAGAACAAATCAAATGAACCAAGTCAAAGCCCTTTTGTTCAGACTTTCTTCCACG<br>GCACAAAGGCTGATCTAAAGATTGGAGACTTTATTGAAGTTGGTCTTAACTCAAACTACGGTCAAAGAAA<br>AAATGCGAAATATATCTACCTGACAGCAACTTTGGATGCAGCTATTTGGGGCGCTGAGCTTGCTCTGGGG<br>GAAGGACGCGAAAGAATCTATTTAGTAGAACCGACAGGGCCAATTGAAGACGACCCCAATTTAACTGATA<br>AAAAATTCCCGGGTAATCCAACAAAATCTTATCGTTCAAAACATCCGTTTAAGGTTGTCGGCGAGATTACT<br>ATTTGGCAAGGACACTCACCCGAACAAGTCAAAGCAATGAATGAGGGATTAGCAAAACTTAAAGAACAA<br>GGTATTGAGGCAATAGAGGAT
Arr-wd2	QTNKTENKSNEPSQSPFVQTFFHGTKADLKIGDFIEVGLNSNYGQRKNAKYIYLTATLDAAIWGAELALGEGR<br>ERIYLVEPTGPIEDDPNLTDKKFPGNPTKSYRSKHPFKVVGEITIWQGHSPEQVKAMNEGLAKLKEQGIEAIED<br>LEHHHHHH (155 aa, 17.5 *k*Da, 18450 $cm^{-1}M^{-1}$)
arr-wd3	GACAAGACCGCCGATGATGCCCGAACCGCGCAGCCGTTCTACCACGGCACGAAGGCGGACCTGAAGCC<br>GGGCGACCTCATCGCGCCGGGCTACGCCTCCAACTACGGCGCGCGGAAGAAAGCAGCCTTCGTCTACCT<br>CACGGCCACGCTCGACGCCGCCACCTGGGGCGCCGAGCTGGCCGCCGGCGAGGGCCGCGGCCGGATC<br>TACTTGGTGGAGCCGACGGGCCCCATCGAGGACGACCCCAACCTCACGGACAAGCGGTTCCCGGGCAA<br>CCCCACGAAGTCGTATCGCTCGCGGGACCCGCTGCGGGTCACGGGCGAGGTCACGGACTGGCAGGGGC<br>ACCCGCACGAGCAGTTGCAGGCCATGCGGGAGCACCTGGAGCGCCTCCGCGCGCAGGGCATCGAGGC<br>GATCGAGGAC
Arr-wd3	DKTADDARTAQPFYHGTKADLKPGDLIAPGYASNYGARKKAAFVYLTATLDAATWGAELAAGEGRGRIYLVE<br>PTGPIEDDPNLTDKRFPGNPTKSYRSRDPLRVTGEVTDWQGHPHEQLQAMREHLERLRAQGIEAIEDLEHH<br>HHHH (147 aa, 16.4 *k*Da, 19940 $cm^{-1}M^{-1}$)
arr-wd4	GAATTTAGCCCGAACAATAATGTTGTCAAACTCTGTCTTCAAGGTATGGAGTTGGAAGAGAAAGGAAA<br>GCCTGAAGAAGCGAGCGAAATATTTCTTCAAGCGTGGAACGAAGCGGCTGACGATTTTGAAAAATTTA<br>CTGCCGGTTATTATGTGGCGCGTCATCAAAACGATACTGCCGACAAATTAAAATGGCTGGAAACGGTTT<br>TGGAGTTCGGATTAAAGATCAATGACGACACGGTTAAGTCGGCTTTTCCCGCTTTGTATCAAAACCTTG<br>CCAAATGCTTTGAGGATTTAGGCGATGCCCGCAACGCGAAAAAGAATTATGAATTAGCAAATTCATTCA<br>AGAAAAAACCGTCCGACAAAGGTCCTTTTTATCACGGAACGCGAGCGGATTTGCAAATCGGCGATTTG<br>CTGACCGCCGGAGGCAAGTCTAATTACAAAGCCGAACTCGTAATGAATCACATTTACTTTACGGCATTG<br>GTGAACGGCGCGGGACTTGCCGCGGCTTTGGCTAACGGCGACGGACGCGAGCGGGTTTATATAGTCG<br>AACCGACGGGGAGTTTTGAAAATGATCCGAATGTGACGGACAAAAAATTTCCGGGCAATCCGACACGC<br>TCATATCGCAGTCAAGCACCTTTGAAAATCGTCGGCGAAGTAACAGATTGGGTGAGAATAACGCCTGA<br>GGAACTCCAAAAATGGCGGGAAAAGTTGGCGAACAGCAAAGGAGAAATTATTAAT
Arr-wd4	EFSPNNNVVKLCLQGMELEEKGKPEEASEIFLQAWNEAADDFEKFTAGYYVARHQNDTADKLKWLETVLEF<br>GLKINDDTVKSAFPALYQNLAKCFEDLGDARNAKKNYELANSFKKKPSDKGPFYHGTRADLQIGDLLTAGGKS<br>NYKAELVMNHIYFTALVNGAGLAAALANGDGRERVYIVEPTGSFENDPNVTDKKFPGNPTRSYRSQAPLKIV<br>GEVTDWVRITPEELQKWREKLANSKGEIINLEHHHHHH (254 aa, 28.6 *k*Da, 35410 $cm^{-1}M^{-1}$)

Table S4. Primer sequences used for the current work

template protein	mutations	sequences
Arr-wd3	N34H<br>(Arr-wd3+N34H)	5'-G GGC TAT GCA AGC CAT TAT GGT GCA CG-3'<br>5'-C GTG CAC CATA ATG GCT TGC ATA GCC C-3'
Arr-wd3	H125M<br>(Arr-wd3+H125M)	5'-GCA ATG CGC GAA ATG CTG GAA CGC CTG-3'<br>5'-CAG GCG TTC CAG CAT TTC GCG CAT TGC-3'
Arr-wd3	H125G<br>(Arr-wd3+H125G)	5'-CAA TGC GCG AA GGC CTG GAA CGC CTG-3'<br>5'-CA GGC GTT CCA GGC CTT CGC GCA TTG-3'
Arr-wd3	H125K<br>(Arr-wd3+H125K)	5'-GCA ATG CGC GAA AAA CTG GAA CGC CTG-3'<br>5'-CAG GCG TTC CAG TTT TTC GCG CAT TGC-3'
Arr-wd3	H142 to stop codon<br>(Arr-wd3+Δ6His)	5'-GAA GCA ATT GAA GAT TAA GAG CAC CAC CAC-3'<br>5'-GTG GTG GTG CTC TTA ATC TTC AAT TGC TTC-3'

Table S5. ADP-ribosylated product analysis by ESI-MS in negative mode

substrate	Product*	calculated m/z	detected m/z
rifampin	ADP-ribosylrifampin	$[M+H^+]^-$ = 1362.5<br>$[M+Na^+]^-$ =1384.5	1362.8<br>1385.0
rifapentine	ADP-ribosylrifapentine	$[M+H^+]^-$ = 1416.5	1416.8
rifaximin	ADP-ribosylrifaximin	$[M+H^+]^-$ = 1325.4	1325.8
rifabutin	ADP-ribosylrifabutin	$[M+H^+]^-$ = 1386.5<br>$[M+Na^+]^-$ = 1408.5	1386.8<br>1408.9

*The ADP-ribosylated product includes a phosphate group, which makes the molecule to exhibit -2 charge by itself.

Table S6. Steady-state kinetic parameters of Arr-wd proteins in ADP-ribosylation of rifampin and its analogues

(a) NAD^+

	Arr-wd1	Arr-wd2	Arr-wd3	Arr-wd4
k_{cat} (s^{-1})	0.023(3)	0.14(2)	0.027(2)	0.108(9)
K_M (mM)	0.4(2)	2.3(6)	0.11(4)	0.8 (2)
k_{cat}/K_M (s^{-1} M^{-1})	65(9)	61(11)	246(19)	139(12)

(b) rifampin

	Arr-wd1	Arr-wd2	Arr-wd3	Arr-wd4
k_{cat} (s^{-1})	0.010(2)	0.056(3)	0.0206(2)	0.060(3)
K_M (mM)	0.23(19)	0.48(7)	0.18(6)	0.17(8)
k_{cat}/K_M (s^{-1} M^{-1})	45(8)	118(6)	113(8)	357(37)

(c) rifapentine

	Arr-wd1	Arr-wd2	Arr-wd3	Arr-wd4
k_{cat} (s^{-1})	0.014(1)	0.031(1)	0.010(1)	0.045(3)
K_M (mM)	0.11(5)	0.13(3)	0.07(5)	0.11(4)
k_{cat}/K_M (s^{-1} M^{-1})	128(12)	245(10)	147(17)	419(30)

(d) rifaximin

	Arr-wd1	Arr-wd2	Arr-wd3	Arr-wd4
k_{cat} (s^{-1})	0.0047(4)	0.0165(2)	0.0028(4)	0.086(7)
K_M (mM)	0.11(6)	0.03(0)	0.13(10)	0.28(8)
k_{cat}/K_M (s^{-1} M^{-1})	41(4)	533(8)	22(3)	304(23)

(e) rifabutin

	Arr-wd1	Arr-wd2	Arr-wd3	Arr-wd4
k_{cat} (s^{-1})	0.010(1)	0.033(1)	0.013(2)	0.063(6)
K_M (mM)	0.17(10)	0.24(3)	0.22(10)	0.27(9)
k_{cat}/K_M (s^{-1} M^{-1})	60(8)	142(6)	58(7)	230(21)

Table S7. Substrate dissociation constants (K_D) measured from the intrinsic fluorescence quenching

(a) NAD^+ and $NADP^+$

K_D (mM)	NAD^+	$NADP^+$
Arr-wd1	1.23(4)	2.3(1)
Arr-wd2	2.14(5)	2.33(7)
Arr-wd3	1.67(6)	1.89(8)
Arr-wd4	1.75(7)	1.90(6)
Arr-wd3+Δ6His	1.53(4)	2.03(8)
Arr-wd3+N34H	2.08(3)	2.07(5)
Arr-wd3+H125M	1.39(1)	2.5(1)
Arr-wd3+H125G	1.48(5)	2.1 (1)
Arr-wd3+H125K	1.23(4)	2.54(1)

(b) rifampin analogues

K_D (μM)	rifampin	rifapentine	rifaximin	rifabutin
Arr-wd1	12.8(4)	22.5(8)	8.6(2)	7.8(1)
Arr-wd2	3.5(1)	5.4(2)	5.1(2)	5.1(2)
Arr-wd3	25.4(4)	30.6(4)	36.2(9)	34.0(14)
Arr-wd4	1.2(1)	1.7(1)	1.4(1)	1.9(1)
Arr-wd3+Δ6His	12.7(1)	19.4(4)	20.5(6)	20.1(4)
Arr-wd3+N34H	7.1(1)	10.1(1)	5.1(1)	7.2(1)
Arr-wd3+H125M	8.9(3)	13.1(1)	7.0(2)	6.9(2)
Arr-wd3+H125G	5.9(2)	9.5(1)	6.3(2)	8.0(2)
Arr-wd3+H125K	12.8(5)	20.6(6)	11.1(1)	18.1(5)

Table S8. Steady-state kinetic parameters of Arr-wd3 variants in ADP-ribosylation of rifampin analogues

(a) NAD^+

variants	k_{cat} (s^{-1})	K_M (mM)	k_{cat}/K_M (s^{-1} M^{-1})
Arr-wd3	0.027(2)	0.11(4)	246(19)
+ N34H	0.012(1)	0.3(1)	39(4)
+ H125M	0.016(2)	0.20(12)	81(12)
+ H125G	0.018(3)	0.84(30)	21(3)
+ H125K	0.024(2)	0.41(10)	57(5)

(b) rifampin

variants	k_{cat} (s^{-1})	K_M (mM)	k_{cat}/K_M (s^{-1} M^{-1})
Arr-wd3	0.0206(2)	0.18(6)	113(8)
+ N34H	0.0077(2)	0.011(5)	702(15)
+ H125M	0.026(1)	0.06(2)	417(21)
+ H125G	0.018(1)	0.09(4)	207(16)
+ H125K	0.0143(5)	0.06(1)	227(7)

(c) rifapentine

variants	k_{cat} (s^{-1})	K_M (mM)	k_{cat}/K_M (s^{-1} M^{-1})
Arr-wd3	0.010(1)	0.07(5)	147(17)
+ H125M	0.0214(10)	0.05(2)	409(19)
+ H125G	0.018(1)	0.08(3)	229(14)
+ H125K	0.0180(6)	0.06(1)	305(9)

(d) rifaximin

variants	k_{cat} (s^{-1})	K_M (mM)	k_{cat}/K_M (s^{-1} M^{-1})
Arr-wd3	0.0028(4)	0.13(10)	22(3)
+ H125M	0.0238(9)	0.09(2)	277(11)
+ H125G	0.0240(9)	0.07(2)	354(13)
+ H125K	0.0205(6)	0.10(1)	204(6)

(e) rifabutin

variants	k_{cat} (s^{-1})	K_M (mM)	k_{cat}/K_M (s^{-1} M^{-1})
Arr-wd3	0.013(2)	0.22(10)	58(7)
+ H125M	0.0138(6)	0.05(2)	288(13)
+ H125G	0.0226(7)	0.09(2)	249(8)
+ H125K	0.0154(2)	0.06(1)	241(3)

Table S9. Determination of minimum inhibitory concentration (MIC) of rifampin and its analogues

(a) MIC values from the literatures (1).

plasmids	IPTG	rifampin	rifaximin	rifabutin
pET28a(+)	-	4	8	8
	+	8	16	8
pET28a(+)-arr-2	-	512	>512	>512
	+	>512	>512	>512
pET22b(+)	-	8	16	16
	+	8	16	16
pET22b(+)-arr-*ms*	-	128	>512	64
	+	>512	>512	>512
pET15b(+)	-	8	16	16
	+	8	16	16
pET15b(+)-arr-*sc*	-	128	>512	64
	+	512	512	512

(b) Measured MIC values in this study

plasmids	IPTG	rifampin	rifapentine	rifaximin	rifabutin
pET22b(+)	-	4	4	4	4
	+	2	4	4	4
pET22b(+)-arr-wd1	-	4	4	4	4
	+	32	64	512	64
pET22b(+)-arr-wd2	-	32	64	256	32
	+	512	>512	>512	256
pET22b(+)-arr-wd3	-	8	8	16	16
	+	64	128	512	128
pET22b(+)-arr-wd4	-	8	16	32	8
	+	128	256	>512	256
pET22b(+)-arr-wd3+N43H	-	8	8	16	8
	+	32	64	>512	128
pET22b(+)-arr-wd3+H125M	-	8	8	16	8
	+	32	64	512	128
pET22b(+)-arr-wd3+H125G	-	16	8	64	16
	+	64	128	>512	128
pET22b(+)-arr-wd3+H125K	-	8	16	16	8
	+	16	32	512	64

Figure S1. Structural analysis of Arr-*ms* protein complexed with rifampin (2HW2.pdb). (a) Annotated secondary structure information and rifampin binding loop (b) 2D diagram of rifampin-Arr-*ms* interaction (color scheme: pink and purple: hydrophobic interaction, green: van der Waals contact, lightblue: hydrogen bond, orange: cation-pi interaction) (c) Water mediated hydrogen bonds between rifampin and Arr-*ms*

Figure S2. Autodock simulation of NAD^+-binding site in Arr-*ms*

Figure S3. Protein purification and characterization of Arr-wd proteins. (a) A representative FPLC trace of Arr-wd1 protein (b) 15% SDS-PAGE of Arr-wd proteins and the single variants (c) A representative MADLI-TOF spectra of Arr-wd1 protein

Figure S4. A representative HPLC analysis of steady-state activity assay by the (a) previously reported HPLC method and by the (b) modified quenching method (c) The standard curve of nicotinamide (d-e) Michaelis-Menten kinetic analysis under various concentrations of (d) NAD^+ or (e) rifampin

Figure S5. Intrinsic fluorescence changes upon the addition of either NAD^+ or rifampin. (a) Relative location of the tryptophan residues in Arr-ms (PDB 2HW2) (b) UV-Vis spectra of rifampin and NAD^+. Rifampin exhibits strong absorption at 295 and 325 nm. (c) Representative intrinsic fluorescence quenching upon the addition of rifampin. The fluorescence intensity was corrected for the secondary filter effect with rifampin as shown in (b). (d) Plots of F_o/F vs the concentration of rifampin. (e) Dissociation constant (K_D) determined by the fluorescence changes **Figure S6.** Rifampin analogues used for the current study

Figure S7. Representative HPLC traces of the reactions with rifampin analogues. (a) rifapentine (b) rifaximin (c) rifabutin

Figure S8. MIC values of Arr-wd proteins. (a) Arr-wd 1-4 proteins (b) Arr-wd3 variants

Figure S1. Structural analysis of Arr-ms protein complexed with rifampin (2HW2.pdb). (a) Annotated secondary structure information and rifampin binding loop (b) 2D diagram of rifampin-Arr-*ms* interaction (color scheme: pink and purple: hydrophobic interaction, green: van der Waals contact, lightblue: hydrogen bond, orange: cation-pi interaction) (c) Water mediated hydrogen bonds between rifampin and Arr-*ms*.

(a)

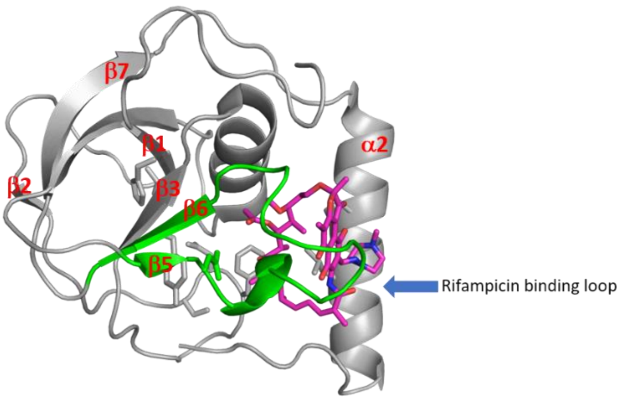

(b)

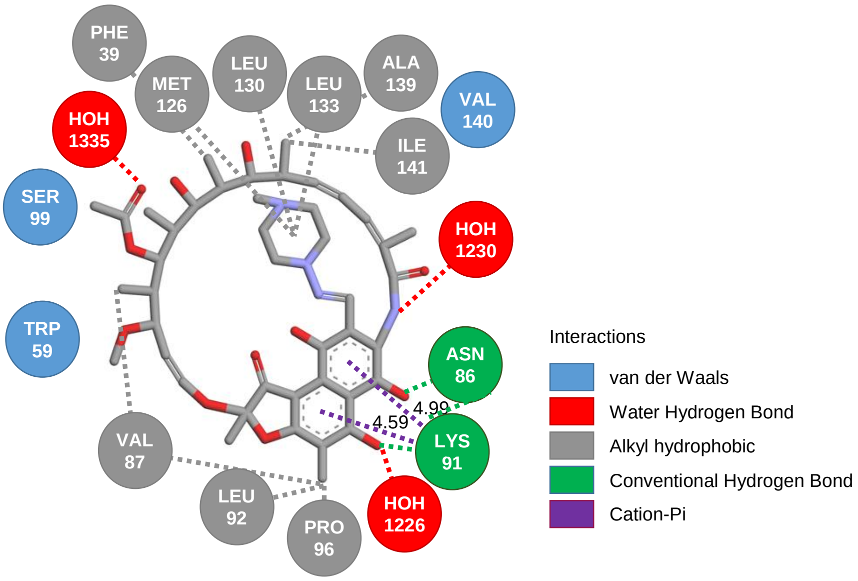

(c)

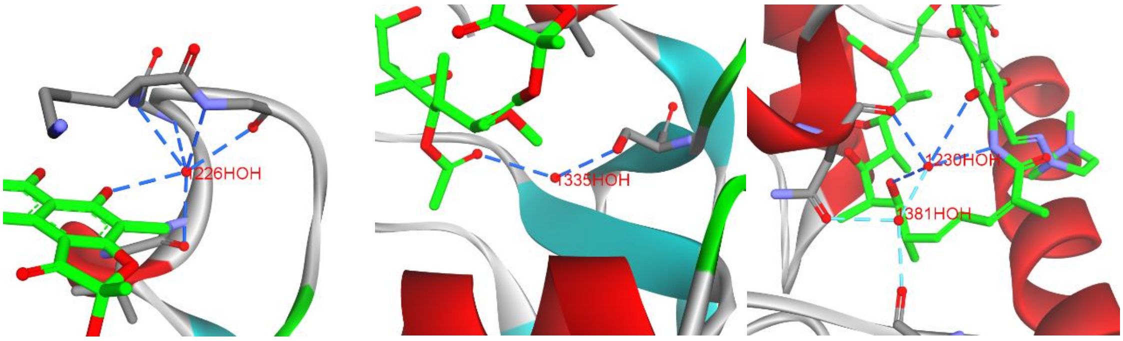

Figure S2. Autodock simulation of NAD$^+$-binding site in Arr-*ms*. The black arrows indicate the scissile C-N bond in NAD$^+$ that generates an oxocarbenium ion in accordance with the release of nicotinamide.

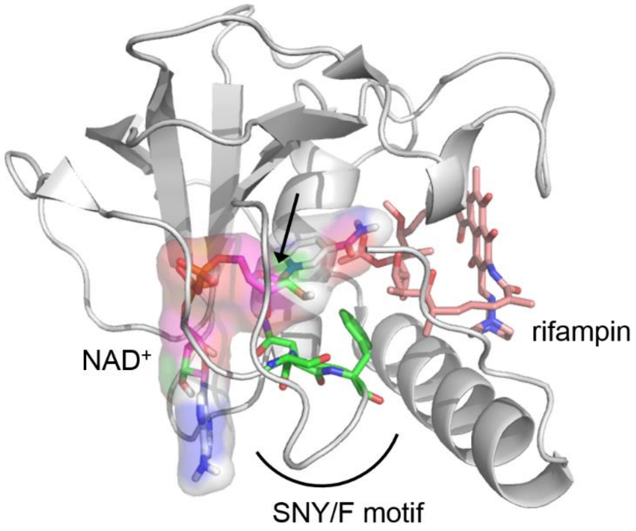

Figure S3. Protein purification and characterization of Arr-wd proteins. (a) A representative FPLC trace of Arr-wd1 protein (b) 15% SDS-PAGE of Arr-wd proteins and the single variants (c) A representative MADLI-TOF spectra of Arr-wd1 protein.

(a)

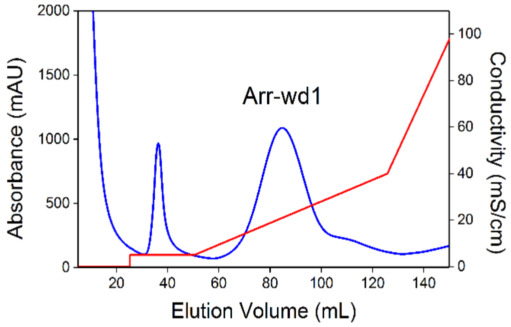

(b)

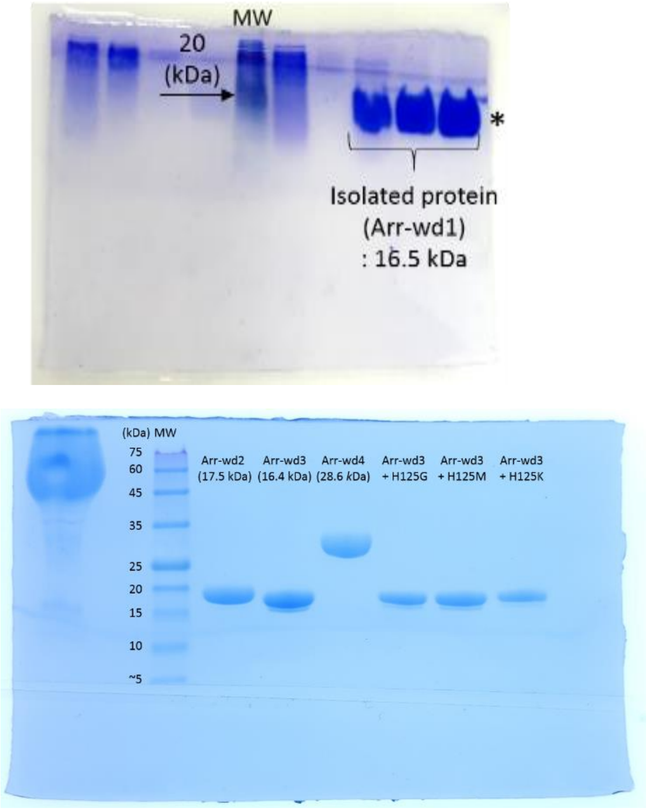

(c)

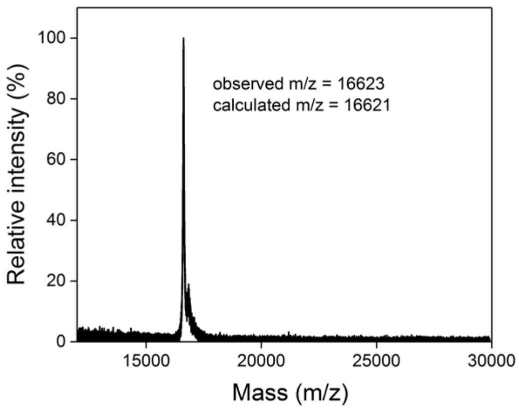
Relative intensity (%)
100
80
60
40
20
0
observed m/z = 16623
calculated m/z = 16621
15000
20000
25000
30000
Mass (m/z)

Figure S4. A representative HPLC analysis of steady-state activity assay. (a) Previously reported HPLC method (b) Modified quenching method by increasing the volume of methanol from 100 µL to 400 µL. (c) The standard curve of nicotinamide by the modified HPLC method (d-e) Michaelis-Menten kinetic analysis under various concentrations of (d) NAD^+ or (e) rifampin.

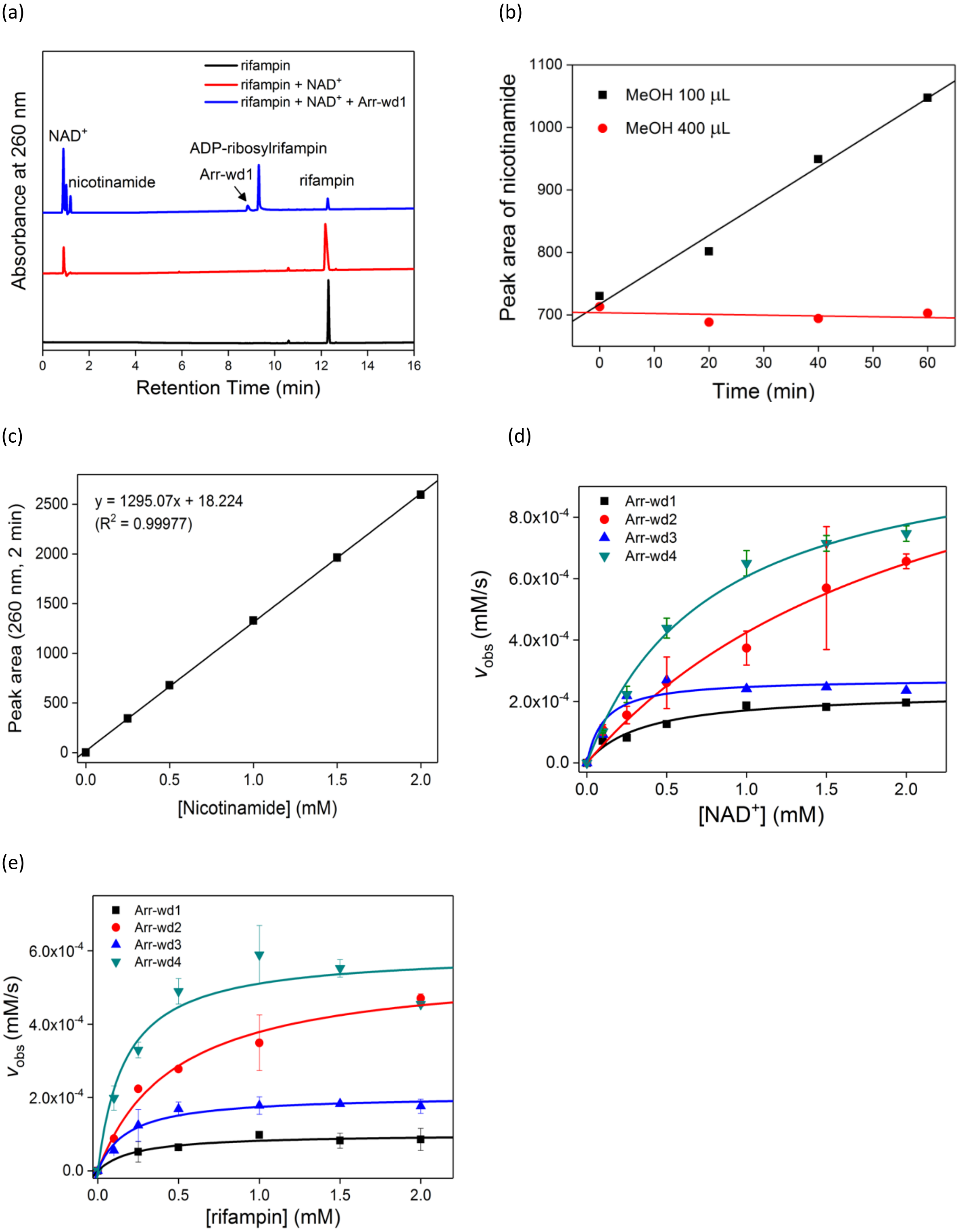

Figure S5. Intrinsic fluorescence changes upon the addition of either NAD^+ or rifampin. (a) Relative location of the tryptophan residues in Arr-ms (PDB 2HW2) (b) UV-Vis spectra of rifampin and NAD^+. Rifampin exhibits strong absorption at 295 and 325 nm. (c) Representative intrinsic fluorescence quenching upon the addition of rifampin. The fluorescence intensity was corrected for the secondary filter effect with rifampin as shown in (b). (d) Plots of F_o/F vs the concentration of rifampin. (e) Dissociation constant (K_D) determined by the fluorescence changes (a)

(b)

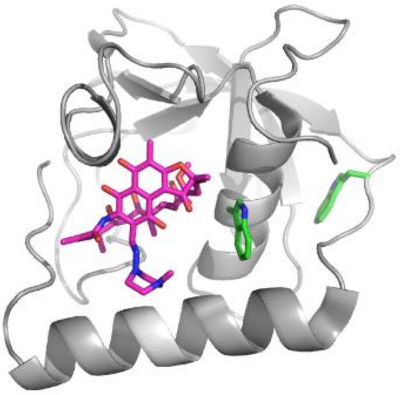

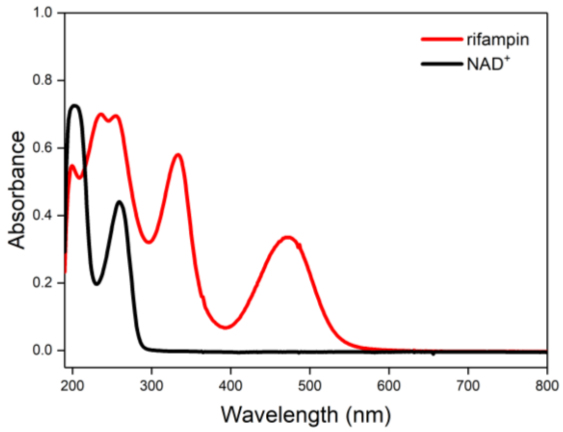

(c)

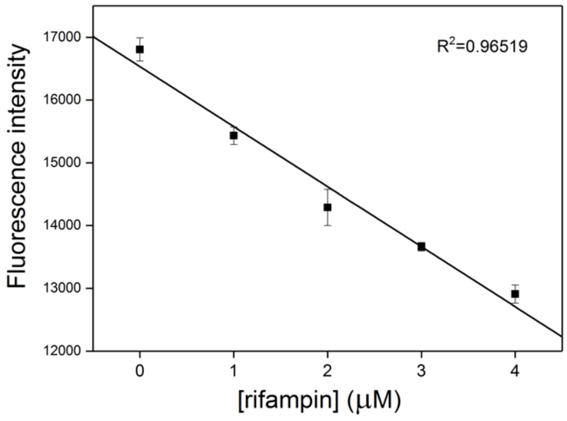

(d)

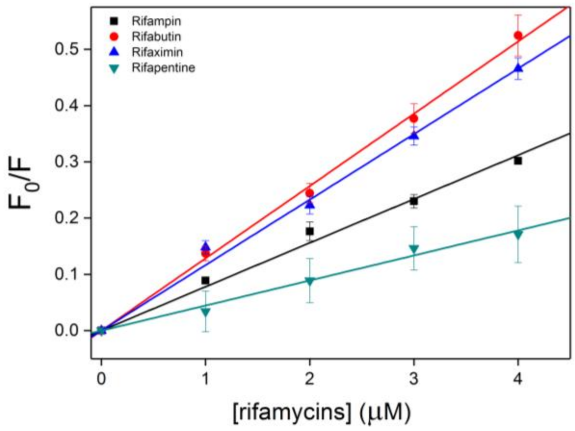

Figure S6. Rifampin analogues used for the current study. Altered substructures of the rifampin derivatives are colored in red, and the hydroxyl group that reacts with an oxocarbenium ion is colored in blue.

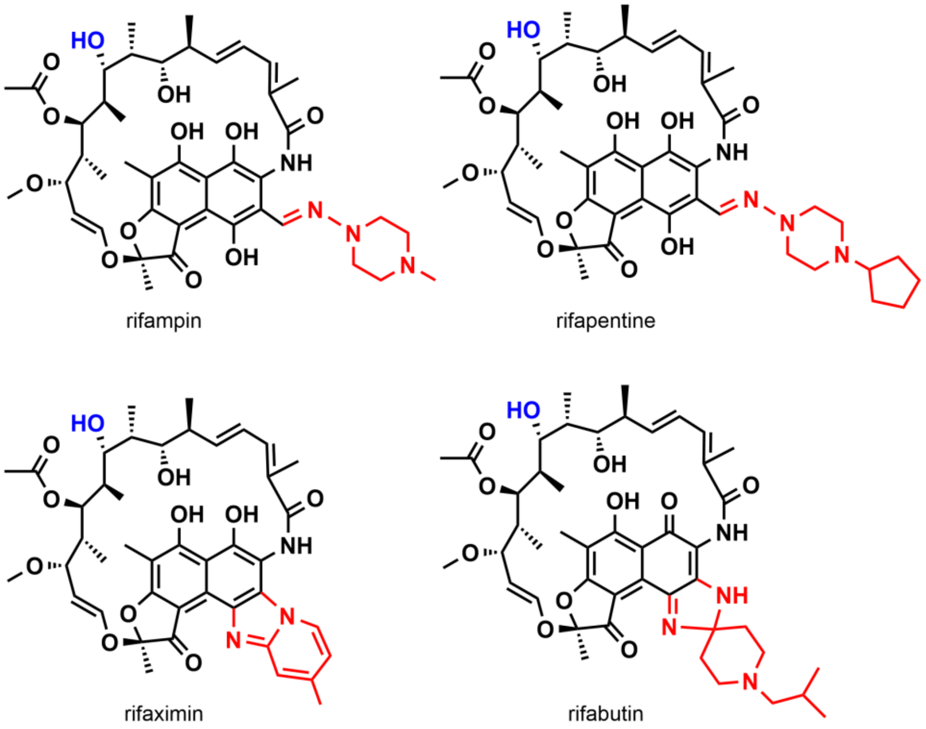

Figure S7. Representative HPLC traces of the reactions with rifampin analogues. (a) rifapentine (b)rifaximin (c) rifabutin.

(a)

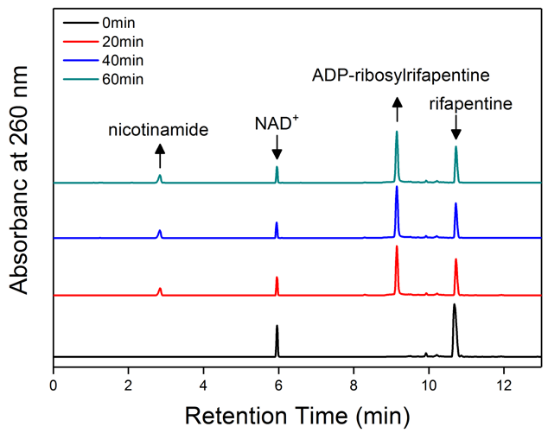

(b)

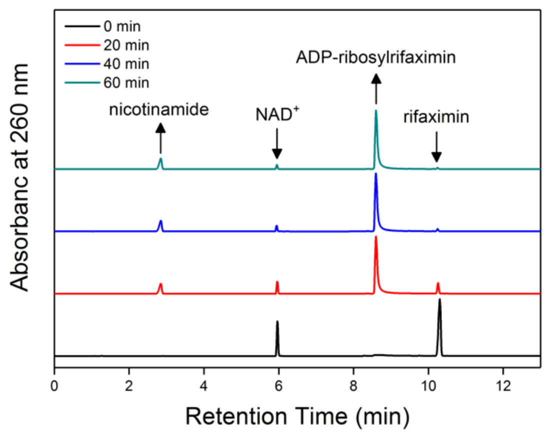

(c)

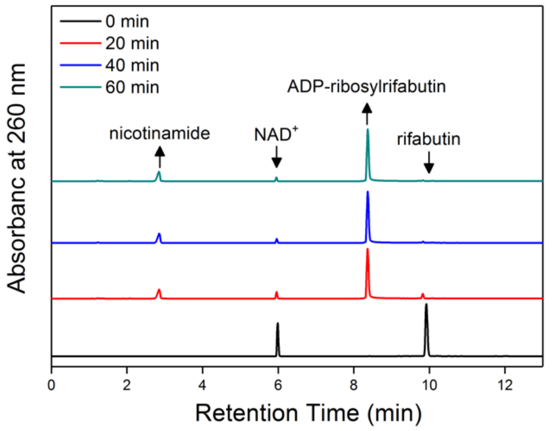

Figure S8. MIC values of Arr-wd proteins. (a) Arr-wd 1-4 proteins (b) Arr-wd3 variants.

(a)

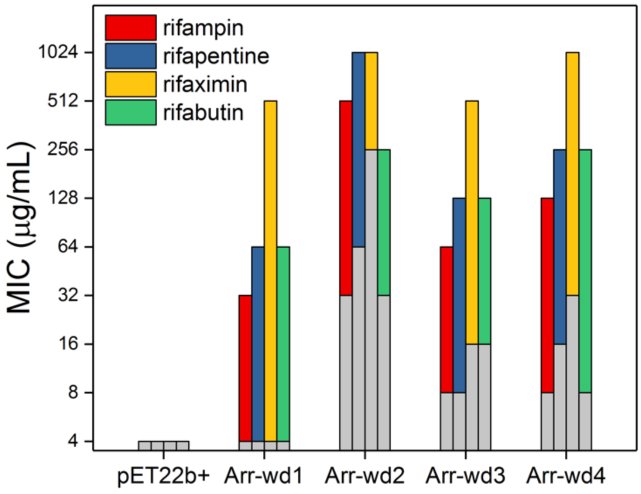

(b)

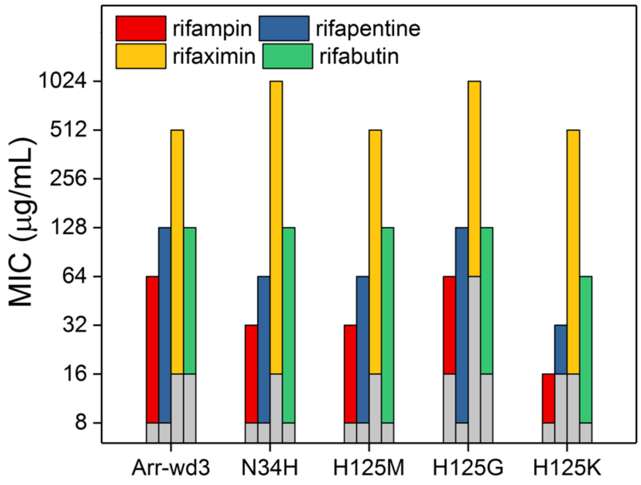

References

1. Baysarowich, J., Koteva, K., Hughes, D.W., Ejim, L., Griffiths, E., Zhang, K., Junop, M. and Wright, G.D. (2008) Rifamycin antibiotic resistance by ADP-ribosylation: Structure and diversity of Arr. *Proc. Natl. Acad. Sci. U.S.A.*, **105**, 4886-4891.
